# Sulfatase-2 from Cancer Associated Fibroblasts: An Environmental Target for Hepatocellular Carcinoma?

**DOI:** 10.1159/000525375

**Published:** 2022-07-13

**Authors:** Marco Y.W. Zaki, Sari F. Alhasan, Ruchi Shukla, Misti McCain, Maja Laszczewska, Daniel Geh, Gillian L. Patman, Despina Televantou, Anna Whitehead, João P. Maurício, Ben Barksby, Lucy M. Gee, Hannah L. Paish, Jack Leslie, Ramy Younes, Alastair D. Burt, Lee A. Borthwick, Huw Thomas, Gary S. Beale, Olivier Govaere, Daniela Sia, Quentin M. Anstee, Dina Tiniakos, Fiona Oakley, Helen L. Reeves

**Affiliations:** ^a^Newcastle University Translational and Clinical Research Institute, Faculty of Medical Sciences, Newcastle University, Newcastle-upon-Tyne, UK; ^b^Department of Biochemistry, Faculty of Pharmacy, Minia University, Minia, Egypt; ^c^Newcastle Fibrosis Research Group, Faculty of Medical Sciences, Newcastle University, Newcastle-upon-Tyne, UK; ^d^Newcastle University Biosciences Institute, Faculty of Medical Sciences, Newcastle University, Newcastle-upon-Tyne, UK; ^e^Department of Cellular Pathology, Newcastle upon Tyne Hospitals NHS Foundation Trust, Newcastle-upon-Tyne, UK; ^f^Department of Medicine, Freeman Hospital, Newcastle-upon-Tyne Hospitals NHS Foundation Trust, Newcastle upon Tyne, UK; ^g^Division of Liver Diseases, Department of Medicine, The Tisch Cancer Institute, Icahn School of Medicine at Mount Sinai, New York, New York, USA; ^h^Department of Pathology, Aretaieion Hospital, National and Kapodistrian University of Athens, Athens, Greece

**Keywords:** Biomarkers, Glypican-3, Hepatocellular carcinoma, Inflammation, Metabolic disease, Molecular targets, NASH, Sulfatase-2, Tumour microenvironment, Spheroids

## Abstract

**Introduction:**

Heparin sulphate proteoglycans in the liver tumour microenvironment (TME) are key regulators of cell signalling, modulated by sulfatase-2 (SULF2). SULF2 overexpression occurs in hepatocellular carcinoma (HCC). Our aims were to define the nature and impact of SULF2 in the HCC TME.

**Methods:**

In liver biopsies from 60 patients with HCC, expression and localization of SULF2 were analysed associated with clinical parameters and outcome. Functional and mechanistic impacts were assessed with immunohistochemistry (IHC), in silico using The Cancer Genome Atlas (TGCA), in primary isolated cancer activated fibroblasts, in monocultures, in 3D spheroids, and in an independent cohort of 20 patients referred for sorafenib. IHC targets included αSMA, glypican-3, β-catenin, RelA-P-ser536, CD4, CD8, CD66b, CD45, CD68, and CD163. SULF2 impact of peripheral blood mononuclear cells was assessed by migration assays, with characterization of immune cell phenotype using fluorescent activated cell sorting.

**Results:**

We report that while SULF2 was expressed in tumour cells in 15% (9/60) of cases, associated with advanced tumour stage and type 2 diabetes, SULF2 was more commonly expressed in cancer-associated fibroblasts (CAFs) (52%) and independently associated with shorter survival (7.2 vs. 29.2 months, *p* = 0.003). Stromal SULF2 modulated glypican-3/β-catenin signalling in vitro, although in vivo associations suggested additional mechanisms underlying the CAF-SULF2 impact on prognosis. Stromal SULF2 was released by CAFS isolated from human HCC. It was induced by TGFβ1, promoted HCC proliferation and sorafenib resistance, with CAF-SULF2 linked to TGFβ1 and immune exhaustion in TGCA HCC patients. Autocrine activation of PDGFRβ/STAT3 signalling was evident in stromal cells, with the release of the potent monocyte/macrophage chemoattractant CCL2 in vitro. In human PBMCs, SULF2 preferentially induced the migration of macrophage precursors (monocytes), inducing a phenotypic change consistent with immune exhaustion. In human HCC tissues, CAF-SULF2 was associated with increased macrophage recruitment, with tumouroid studies showing stromal-derived SULF2-induced paracrine activation of the IKKβ/NF-κB pathway, tumour cell proliferation, invasion, and sorafenib resistance.

**Conclusion:**

SULF2 derived from CAFs modulates glypican-3/β-catenin signalling but also the HCC immune TME, associated with tumour progression and therapy resistance via activation of the TAK1/IKKβ/NF-κB pathway. It is an attractive target for combination therapies for patients with HCC.

## Introduction

Liver cancer is the fifth most common cancer and the second cause of cancer-related mortality worldwide [1]. Hepatocellular carcinoma (HCC) accounts for 80–90% of cases, typically developing in the presence of chronic liver disease (CLD) caused by hepatotropic viruses, alcohol excess, or obesity [2]. DNA damage and molecular aberrations in hepatocytes contribute to cancer development, but tumour heterogeneity and failures to identify genetic drivers common to large numbers of patients have hampered targeted therapeutic advances. The tumour microenvironment (TME), comprising endothelial, mesenchymal, and immune cells, secreted extracellular matrix proteins and growth factors, can also influence tumour biology and progression [3]. Therefore, common TME modulators may be worthy therapeutic candidates. Indeed, targeting the immune cell checkpoints has recently heralded a major advance in the treatment of HCC, although as yet, not all patients respond [4]. Additional TME candidates, to target alone or in combination, are eagerly awaited.

Mature active sulfatase-2 (SULF2), which can be membrane bound or secreted, is an endo-sulfatase that removes sulphate groups at the 6-O position on sugars in heparansulfate (HS) chains of HS proteoglycans (HSPGs) [5]. In a number of cancers, SULF2 upregulation is associated with more advanced disease and poorer patient outcome [6, 7, 8]. Conversely, supressed SULF2 expression has been reported to predict higher sensitivity to topoisomerase-I inhibition in patients with lung cancer [9]. Previous microarray gene expression studies have identified elevated SULF2 mRNA levels in 79 (57%) of 139 HCCs, as well as 8 (73%) of 11 HCC cell lines [10]. Patient studies were in surgical candidates, where elevated SULF2 was associated with modulation of glypican-3 and fibroblast growth factor signalling, a higher rate of recurrence and shorter survival [10]. Expression in patients with more advanced disease is not well studied. The cellular source of SULF2 in patients with HCC, its targets, its impact on the HCC TME, and patient outcome remain to be comprehensively characterized.

## Materials and Methods

### Patients

The retrospective case series included 60 patients with HCC presenting to the Newcastle upon Tyne Hospitals NHS Foundation Trust between 2000 and 2010, with surplus formalin-fixed, paraffin-embedded (FFPE) tissues available for research, as well as *n* = 20 later patients treated with sorafenib. Patients with histologically benign disease or cholangiocarcinoma were excluded. Patient demographics and clinicopathological information included age, gender, underlying liver disease aetiology, liver function, Edmondson-Steiner tumour grade [11] modified for biopsy reporting by experienced pathologists, tumour node metastases and combined Barcelona Clinic for Liver Cancer (BCLC) stages [12], treatments administered, and patient survival from the time of biopsy. In an additional cohort of 20 patients with advanced disease referred for sorafenib, responders were those who had stable disease on imaging for at least 3 months, while non-responders were those whose treatment was discontinued due to poor tolerance or disease progression on imaging at 3 months. Finally, primary culture cancer activated fibroblasts (CAFs) were derived from human HCC tissues in 2021 (online suppl. Methods; for all online suppl. material, see www.karger.com/doi/10.1159/000525375).

### Tissues and Modelling Studies

FFPE diagnostic biopsy tissue sections containing both HCC and non-neoplastic liver parenchyma were immunostained for SULF2, αSMA, glypican-3, RelA-P-ser536, CD45, CD4, CD8, CD66b, CD68, and CD163, scanned and visualized with Aperio Imagescope Software. Two pathologists, blinded to patient outcome, assessed SULF2-immunostained slides. SULF2 in tumour cells was graded as absent or present, with present indicated by ≥5% of tumour cells having positive cytoplasmic immunostaining. SULF2 in CAFs was graded as absent or present, where “absent” included cases with either no or scant SULF2 and “present” included cases with intense focally positive or diffusely positive SULF2. Glypican-3 in tumour cells was graded as 0–3, corresponding to absent (0), cytoplasmic dot like or focal positivity [1], more diffuse weak positivity in cytoplasm or membrane [2] and intensely positive membranous or cytoplasmic staining [3]. RelA-P-ser536 was scored as absent, present in scattered tumour nuclei, present in >50% nuclei, and present in >90% nuclei.

Biopsies from the cohort of sorafenib-treated patients were additionally stained for CD45 (pan immune marker), CD4, CD8 (T cell markers), CD66b (neutrophil marker), and CD68 and CD163 (macrophage markers). The IHC slides were analysed digitally using Aperio Imagescope, with a positive pixel algorithm expressed as a ratio of total negative + positive pixels.

### Cell Culture

LX-2 liver myofibroblast cells were a gift of Scott Freidman (Mount Sinai, USA) and were cultured in DMEM high glucose media supplemented with 1% penicillin and streptomycin, L-glutamine, and 2% FBS. These cells were validated with 100% match to a profile for LX-2 CVCL_5792, comparing 8 sort tandem repeat loci in the Cellosaurus Database, by NorthGene Limited, Newcastle, UK. Huh7 and Hep3B HCC cells were purchased from the American Type Culture Collection (ATCC), using stocks frozen within 6 passages from purchase. COS-7 fibroblasts were a gift from Ralf Weiskirchen (Aachen, Germany). These were confirmed to be of African Green Monkey origin by mitochondrial DNA PCR using species-specific primers, with sort tandem repeat analysis showing an 87.5% match (7/8 loci) with the previously reported CV1/COS-7 profile [13]. COS-7 cells were cultured in DMEM high glucose media supplemented with 1% penicillin and streptomycin, L-glutamine and 10% FCS. All cells were cultured at 37°C in an atmosphere of 5% CO_2_. LX-2 cells were stimulated with 10 ng TGFβ for 48 h unless stated otherwise.

### Stable SULF2 Knockdown

Huh7 HCC cells and COS-7 cells were transduced with mission TRC2 shRNA lentiviral particles targeting SULF2 (TRCN0000364518; Sigma-Aldrich, USA) or TRC2-pLKO-puro nontargeting (NT) with hexadimethrine bromide (shRNA sequences in online suppl. Table 1). Cells were selected using puromycin.

### 3D Tumour Spheroid Hanging Droplets

*Mixed cell-type spheroids*; 1,500 cells from Huh7 or Hep3B HCC cells were combined 1:1 with control or SULF2 knockdown (KD) COS-7 cells in 20 µL media per sphere on the lid of a 10 cm^3^dish (*n* = 10 per group). The lid was inverted and cells were gravity suspended in hanging droplets for 3 days with 10 mL sterile PBS in the dish beneath to maintain a humidified environment. The change in spheroid volume from days 3–8 was quantified. *Tumour cell spheroids*; 3,000 Huh7 or Hep3B cells were suspended in hanging droplets to form spheres. Spheroids were treated with stromal conditioned media (CM) for 3 days. Spheroid volume was calculated by measuring the area and applying the formula *V*_mm3_ = 0.09403 × ((*A*_pixel_ × 0.28)/1,000)^1.5^.

Details of in vitro cell culture, 2D and 3D models, SULF2 KD, CCL2 and SULF2 ELISA, primary CAF isolation, migration assays, fluorescent activated cells sorting, RNA extraction, Western blotting, quantitative RT-PCR, primer sequences and antibodies are provided in the supplementary information and online supplementary Tables 1–3.

### Statistics

Statistical analyses were conducted using SPSS version 23 or GraphPad Prism version 7.00. For comparisons between groups, continuous data was assessed with Mann-Whitney or Kruskall-Wallis tests. Categorical data was analysed using χ^2^ tests. The principal documented endpoint in the clinical cases studied was overall survival, recorded as months from diagnosis until January 31, 2019. Differences in cumulative survival were determined using the Kaplan-Meier method and a Log-Rank test. The Cox proportional hazards-regression model was used to identify parameters associated with survival.

## Results

### Elevated SULF2 in the HCC TME Was Associated with Poorer Survival

SULF2 tissue expression patterns were determined immunohistochemically in 60 human HCC diagnostic biopsies. The cohort median age was 69 years with a male to female ratio of 5 to 1. Overall, 49% (29/60) had type 2 diabetes, with 73% of patients having either alcohol-related liver diseases, nonalcoholic fatty liver diseases, or no known CLD. Cirrhosis was absent in 49% (29/60), reflecting the need for biopsy rather than radiological diagnosis in the absence of established cirrhosis. This selected cohort was of patients deemed fit for therapy and the majority had preserved liver function and BCLC A-C disease, with an overall median survival of 20.3 months. Demographic and clinicopathological features are summarized in Table 1.

**Fig. 1:**
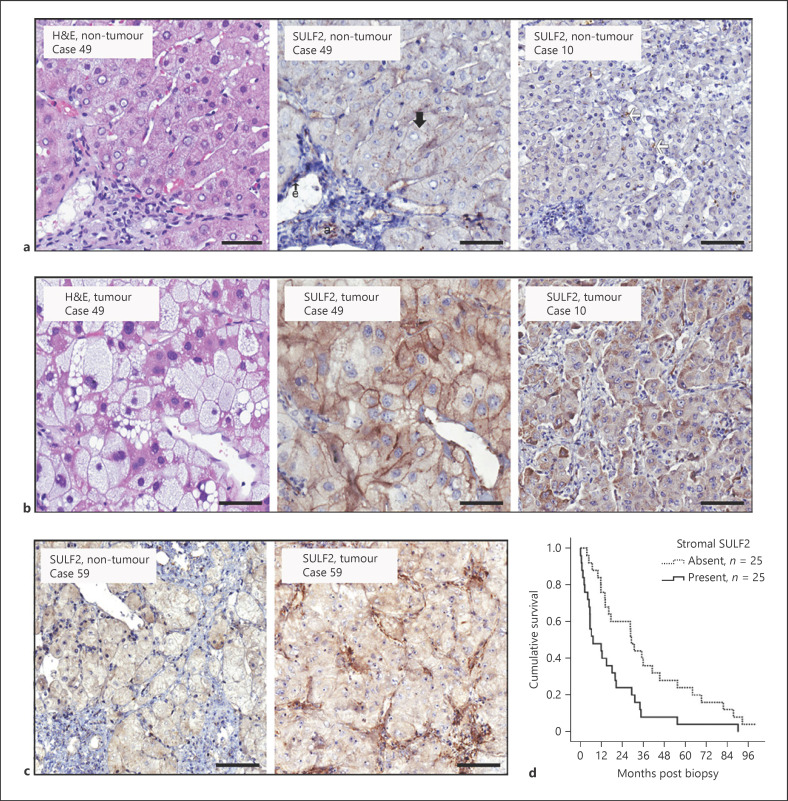
SULF2 expression in non-tumour liver and HCC tumour cells predicts prognosis: Representative images show H&E staining and SULF2 IHC in non-tumour (**a**) and tumour paired tissues (**b**). In non-tumour liver, SULF2 was expressed on the canalicular surface hepatocytes (black arrow), hepatic arterioles (a), endothelial cells (e), and occasional nonparenchymal sinusoidal cells (white arrows). In the tumour, cytoplasmic (right image) and membranous SULF2 (middle image) was increased in neoplastic cells. **c** Images show SULF2 IHC in non-tumour and tumour paired tissue. SULF2 was upregulated in CAFs in tumour tissue as compared to minimal expression in non-tumour stroma. Images are ×20 magnification and scale bars represent 50 microns. **d** Survival of patients with stromal SULF2 present in their tumour was markedly reduced (median 7.2 months) compared to patients in whom it was absent/scanty (median 29.2 months; Kaplan-Meier, *p* = 0.003).

In non-neoplastic and neoplastic tissues, SULF2 was detected in endothelial cells that served as an internal positive IHC control. SULF2 was detected at low levels in hepatic arterioles, benign bile duct epithelium, occasional nonparenchymal cells within the portal tracts and sinusoids, as well as on the canalicular surfaces of hepatocytes (Fig. 1a). In tumour tissues, SULF2 was overexpressed in 58% (35/60) of HCC cases, corroborating previously reported gene expression data. Whole tumour tissue mRNA expression, however, does not capture the predominant cell type responsible for expression. In our biopsy cases, the tumour cells were the source of elevated SULF2 in only 15% (9/60), where SULF2 was detected in the tumour cell cytoplasm and/or membrane (Fig. 1b). The more common cell-type expressing elevated SULF2 were the CAFs-identified αSMA-positive myofibroblast-like cells, with SULF2 expressed in 52% (31/60), in either a focal intense or widespread form (Fig. 1c; online suppl. Fig. 1A). This was corroborated by interrogation of the publicly available (TCGA) human liver cancer dataset on the cBioPortal website (http://www.cbioportal.org/), where SULF2 expression in HCC correlated strongly in a highly significant fashion with the myofibroblast markers vimentin, αSMA (ACTA2), COL1A1, COL1A2, and TIMP2 (online suppl. Fig. 1B). Notably, SULF2 expression was less common and intense in αSMA-positive myofibroblasts in the portal tracts or bridging fibrous septa of the non-neoplastic liver (online suppl. Fig. 1C), in keeping with the TME promoting SULF2 expression.

Clinical features of the nine cases in which SULF2 expression was present versus absent in tumour cells are shown in Table 1. SULF2-positive HCCs were typically larger and associated with extrahepatic disease, consequently with a more advanced tumour node metastasis stage. The alpha-fetoprotein (AFP) level in this small group was also significantly higher (*p* = 0.03). Median survival was reduced, although not significantly so. Elevated SULF2 expression in CAFs demonstrated no specific associations with tumour grade or stage, but survival was similarly reduced in the presence of CAFs-SULF2 upregulation (12.2 months vs. 35.0 months).

Survival analyses excluding surgically treated patients with relatively early-stage HCC (10 treated surgically) highlighted the difference in those with more advanced disease. In the 50 patients receiving locoregional, medical or supportive treatments (Table 1; Fig. 1d), SULF2 elevation in CAFs was associated with a median survival of 7.2 months, compared to 29.2 months in those without CAF-SULF2 (*p* = 0.003; Kaplan-Meier). Univariate analyses to identify factors associated with survival are shown in Table 2, as are those factors with a *p* value <0.05 when entered into a multivariate Cox regression analysis. Serum AFP, albumin level, and performance status, in addition to SULF2 in CAFs, were independently associated with poorer survival (Table 2).

### Glypican-3 in the HCC TME

Glypican-3 is a morphogen HSPG and both its expression and 6-O sulfation, which promote canonical Wnt signalling, have been reported as SULF2 regulated in HCC [10]. Glypican-3 expression is often upregulated in HCC tissues and its IHC detection has been proposed as an HCC histopathology diagnostic biomarker [14, 15]. In our study, there was no glypican-3 expression in non-tumour tissues, but membranous or cytoplasmic expression was present in tumour cells in 44/60 (73%) cases (online suppl. Fig. 2), graded as 0/1/2/3 in 16/17/9/18 cases respectively. In our nonsurgically treated cohort (*n* = 50), glypican-3 expression (absent *n* = 13 or present *n* = 37) was not significantly associated with survival, although intense cytoplasmic or membranous glypican-3 (Grade 3) identified a small group of patients with a particularly poor prognosis (*n* = 14, median survival 6.7 months) (online suppl. Fig. 2B). For cases with Grade 1–2 expression, survival was discriminated by CAF-SULF2 expression (online suppl. Fig. 2D).

### Modelling the Impact of Stromal SULF2

**Fig. 2:**
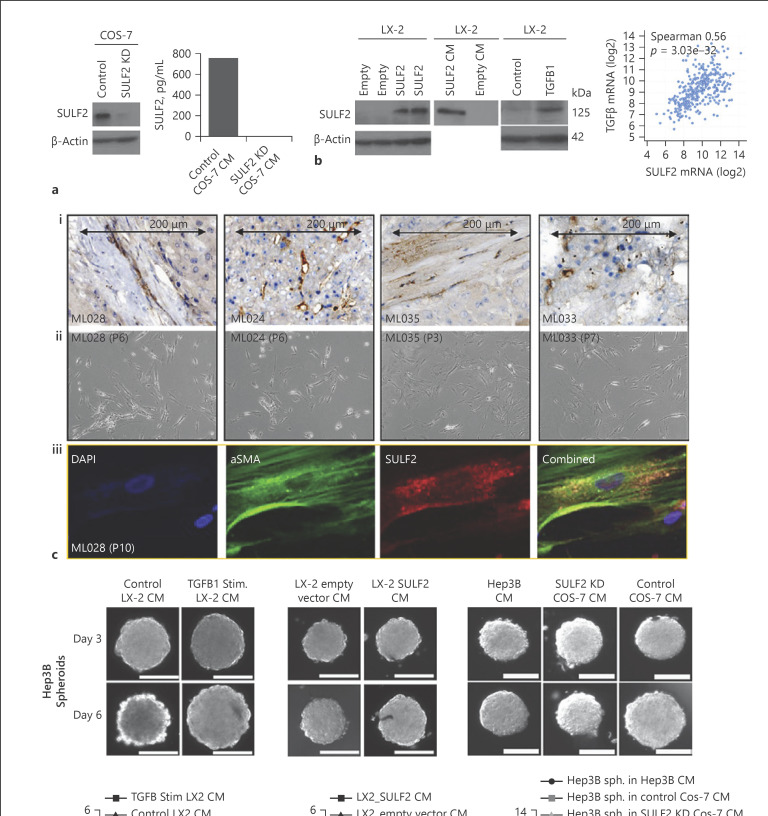
SULF2 is expressed by CAFS isolated from SULF2 stromal positive tumours, with SULF2 conditioned media stimulating growth of 3D Hep3B spheroids in vitro. **a** SULF2 was highly expressed in COS-7 cells and suppressed by SULF2 shRNA, assessed by Western blot and ELISA assay. **b** Western blot of LX-2 whole cell lysates showing LX-2 cells with little SULF2 (left panel). SULF2 expression was induced in LX-2 after transfection with a SULF2 expression vector compared to empty vector control (left). SULF2 levels were elevated in CM from LX-2 cells transfected with a SULF2 expression vector (middle). TGFβ stimulation of LX-2 cells induced SULF2 expression, with a highly significant correlation between mRNA of the two in TCGA dataset (right panels). Mixed cell isolations from SULF2 stromal positive tumours (**ci**) yielded CAFs after trypsinisation and replating in fibroblast culture media (**cii**). **ciii**Dual labelling immunofluorescence confirmed co-expression of SULF2 (red) and αSMA (green) in primary CAFs. CM from TGFβ-stimulated (**d**) or SULF2 expression vector-transfected LX-2 cells (**e**) promoted growth of Hep3B (SULF2 null) spheroids. **f** CM from control COS-7 cells expressing SULF2 promoted spheroid growth in Hep3B cells, as compared to culture with in SULF2 KD COS-7 CM. Scale bars represent 200 microns. Change in spheroid volume is in online supplementary Table 4. Data mean ± SEM; *n* = 7 to 10 spheroids per condition. **p < 0.01; ***p = 0.001; ****p < 0.001. “P” denotes number of passages for CAFs.

To model the impact of stromal SULF2 on tumour cell growth, we used COS-7 cells (African green monkey kidney embryonic fibroblasts expressing high endogenous SULF2, with 98.5% homology to human SULF2; https://www.ensembl.org BLAST [16]), alongside manipulating SULF2 expression in human liver LX-2 myofibroblast cells, which have low levels of endogenous SULF2 (Fig. 2a, b). A stable SULF2 KD was generated in the SULF2-rich COS-7 cells, also confirmed by Western blotting and ELISA (Fig. 2a). SULF2 was transiently overexpressed in LX-2 cells and empty vector-transfected cells as a control (Fig. 2b). Secretion of SULF2 in the media was confirmed by Western blotting (Fig. 2b). The HCC TME, in which we report frequent upregulation of CAF-SULF2, is characterized by activation of the TGFβ1 signalling pathway [17] known to induce SULF2 in renal disease [18]. Indeed, SULF2 protein levels were elevated in LX-2 cells challenged in vitro with exogenous TGFβ1 (Fig. 2b), with a strong and a highly significant correlation in gene expression between the two confirmed in the cancer genome atlas (TCGA) HCC dataset (Fig. 2b). Primary CAFs from 4 human HCC with IHC evidence of stromal SULF2 expression (Fig. 2ci + cii), expressed endogenous *SULF2* and *aSMA*, confirmed by dual labelling immunofluorescence (Fig. 2ciii). SULF2 expression levels have been previously characterized in HCC cell lines [10], with Hep3B cells producing very low levels versus higher expression in Huh7 cells. We compared the impact of SULF2-CM on these low and high expressors in further experiments.

### Stromal SULF2 Promotes the Growth, Migration, and Invasion of HCC Cells in vitro

A 3D-HCC spheroid model was adopted to assess the impact of stromal SULF2 on HCC growth. The growth rate of Hep3B spheroids cultured in CM collected from TGFβ1-stimulated LX-2 cells or cells over-expressing SULF2 was significantly increased compared to spheroids exposed to CM from control unstimulated or empty vector-transfected LX-2 cells, with low SULF2 (Fig. 2d, e). Similarly, Hep3B spheroid growth was significantly increased when cultured in CM from control SULF2-expressing COS-7 cells but not SULF2 KD COS-7 cells (Fig. 2f), confirming an HCC mitogenic role for stromal SULF2.

In a 2D transwell culture, COS-7 cells increased the metabolic activity and cellular proliferation of both Huh7 and Hep3B cells, while tumour cells alone or co-cultured with SULF2 KD COS-7 cells did not (online suppl. Fig. 3A, B). Fibroblast-derived SULF2 also markedly increased the migration and invasion of the SULF2 null Hep3B cell line compared to controls (online suppl. Fig. 3C, D).

### SULF2 Blockade Reversed 3D Spheroid Growth

**Fig. 3:**
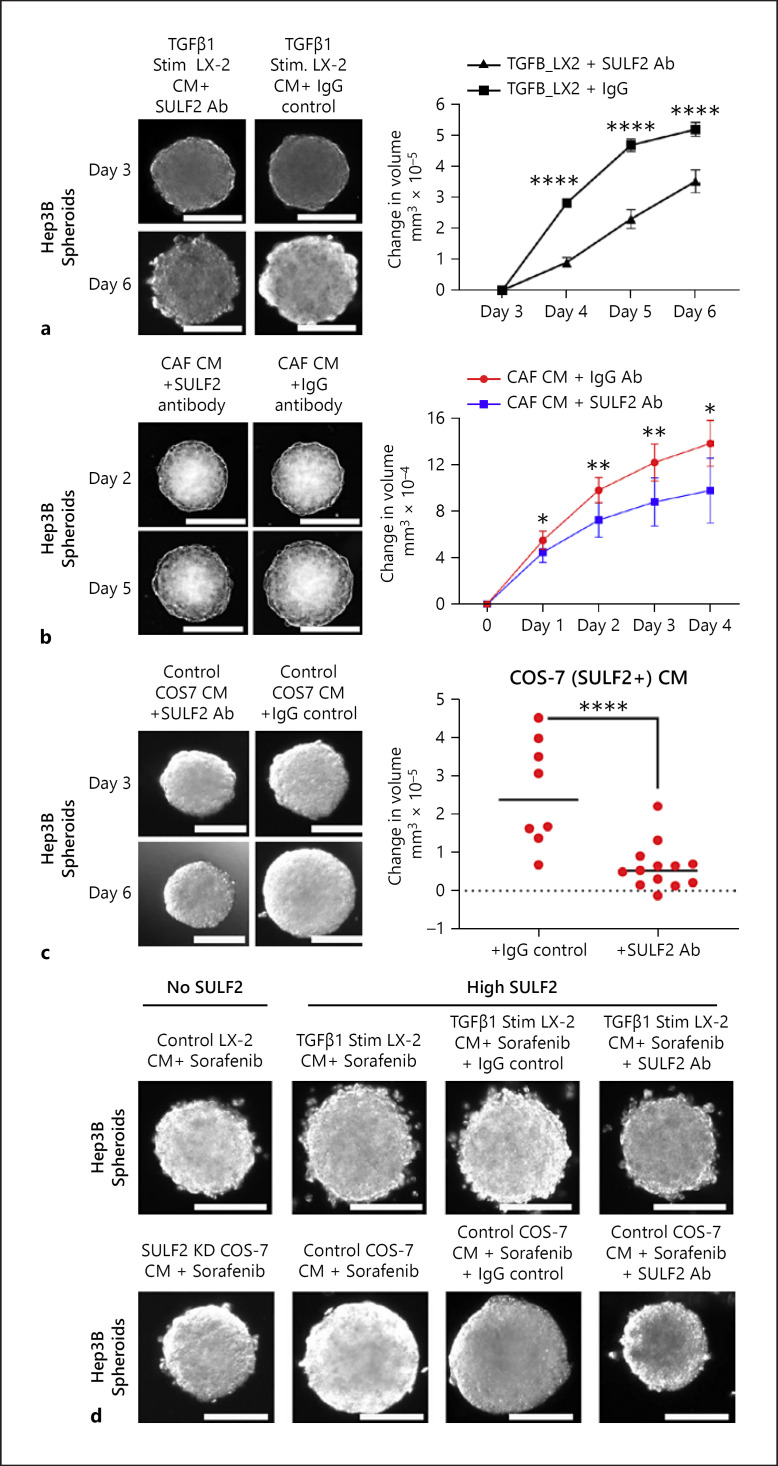
The impact of stromal SULF2 inhibition on the growth of tumour spheroids and SULF2-mediated sorafenib sensitivity: **a**−**c** TGFβ-stimulated LX-2-CM (**a**), primary CAF-SULF2-CM (*n* = 4 SULF2-positive HCC) (**b**), and COS-7 CM (**c**) induced Hep3B spheroid growth was abrogated using SULF2 antibody versus control IgG. **d** Sorafenib treatment had little impact on the growth of Hep3B spheroids cultured in control TGFβ-stimulated LX-2 cell CM (upper images) or COS-7 cell CM (lower images). Growth was dramatically suppressed and sensitivity to sorafenib restored in Hep3B spheroids cultured CM from TGFβ-stimulated LX-2 or SULF2 KD COS-7 cells respectively. Changes in spheroid volume are in online supplementary Table 4. Data mean ± SEM; 7 to 10 spheroids per condition. ****p < 0.001.

Hep3B cell proliferation, induced by CM from TGFβ1-stimulated LX-2 cells or by CM from primary CAFS expressing endogenous SULF2, was suppressed by an anti-SULF2 antibody, rather than an IgG isotype control antibody (Fig. 3a, b). Similarly, COS-7 CM-induced Hep3B proliferation was significantly diminished by an anti-SULF2 antibody but not a control antibody (Fig. 3c). In Huh7 cells that produce SULF2, the SULF2 Ab also suppressed spheroid growth (online suppl. Fig. 4A).

### CAF-SULF2 Contributes to Sorafenib Resistance

For the last decade, sorafenib has been − and remains − a standard first-line therapy for patients with advanced HCC. Sorafenib is associated with a median survival advantage of 2–3 months [19]. In 3D cultures, Hep3B growth was attenuated by sorafenib treatment when cultured in SULF2 low CM from LX-2 cells or SULF2 KD COS-7 cells (Fig. 3d). However, the effect of sorafenib was mitigated, with increased growth observed, when spheroids were grown in CM from TGFβ1-stimulated LX-2 cells or COS-7 cells (Fig. 3d; online suppl. Fig. 4B, C). Treatment with a SULF2 Ab restored sorafenib sensitivity and growth attenuation, supporting SULF2-mediated sorafenib resistance (Fig. 3d; online suppl. Fig. 4B, C).

We explored outcomes in an independent cohort of 20 patients with advanced HCC referred for sorafenib treatment (online suppl. Table 5). Their median age was 72.5 years. The majority had ALD or NAFLD, with 50% having no evidence of cirrhosis. Fourteen were referred for first-line medical therapy, with six referred for 2nd-line treatment, having previously received arterial treatment. Eighteen proceeded with sorafenib treatment, starting at 400 mg twice daily. Seven had a treatment break or dose reduction prior to permanent discontinuation, with sorafenib discontinued either because of toxicity or disease progression. The duration of therapy was shorter in those with focal intense or widespread CAF-SULF2 expression (absent, scant, focal, focal intense, or widespread; Spearman −0.536; *p* = 0.022; *n* = 18 − Kaplan-Meier *p* = 0.003) (online suppl. Fig. 4D).

### Glypican-3 and β-Catenin Expression Were Modulated by CAF-SULF2

**Fig. 4:**
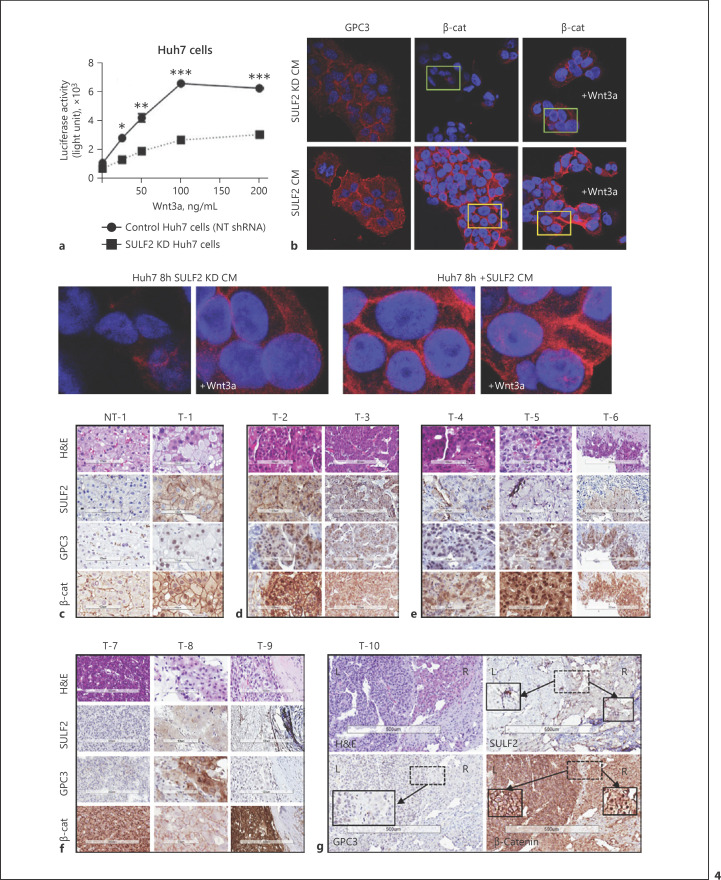
SULF2, Glypican-3 and β-catenin in HCC. In HuH7 cells which overexpress SULF2 (**a**), addition of Wnt3a ligand stimulated a TOPFLASH β-catenin (β-cat) reporter, abrogated by SULF2 KD. **b** Confocal immunofluorescence staining of HuH7 cells showed low levels of glypican-3 (GPC3) and β-catenin in the presence of CM from COS-7 KD cells, +/− Wnt3a 100 ng/mL. In the presence of SULF2-CM, GPC3 and membranous β-cat were upregulated. There was evidence of nuclear β-cat in the presence of Wnt3a (magnified images). **c**−**g** Haematoxylin and eosin images, with SULF2, glypican-3 and β-catenin immunohistochemistry is shown for 10 cases. A non-tumour (NT) case is in (**c**), with the corresponding tumour (T) showing tumour cells SULF2+, nonspecific nuclear GPC3, and nuclear β-cat. Panel (**d**) shows two SULF2+ HCC with grade 1–2 GPC3+ and nuclear β-cat. In (**e**), three CAF-SULF2+ cases show grade 1–2 GPC+ and nuclear β-cat. T7 (**f**), however, has no SULF2 or GCP3, but nuclear β-cat++; T8 has scant SULF2, grade 2 GPC3 and no nuclear β-cat, while T-9 is CAF-SULF2+, GPC3−, and β-cat++. T10 is from a resection, showing tumour heterogeneity, with mild tumour SULF2+ (right [R]), with nuclear β-cat, while a region with CAF-SULF2+ (left [L]) shows membranous accumulation of β-cat. GPC3+ was classed negative, with absent cytoplasmic or membranous stain in both areas.

Mechanistically, we first considered the impact of CAF-SULF2 on canonical Wnt/β-catenin signalling − a well-established target of SULF2. Huh7 cells overexpress SULF2 in the presence of a wild-type Wnt-β-catenin pathway [20]. shRNA SULF2 knockdown Huh7 as well as NT shRNA Huh7 cells were stimulated with the frizzled receptor ligand Wnt3a (100 ng/mL). Wnt3a stimulated the activity of a β-catenin TOPflash luciferase reporter assay in both cell lines, with significantly greater activity in NT shRNA, in keeping with partial dependence on SULF2 (Fig. 4a). Further, in vitro stimulation of Huh7 cells with SULF-CM from COS-7 cells increased GPC3 as well as β-catenin expression, which KD-SULF2-CM did not (Fig. 4b). SULF2-induced β-catenin expression was predominantly membranous. Wnt3a induced nuclear translocation of β-catenin, which was more evident in the SULF-CM-treated cells with higher levels of β-catenin (representative images Fig. 4b − enlarged panels). In our patient cohort, we assessed the relationship between SULF2, glypican-3, and β-catenin by IHC (Fig. 4c-g). While there were cases with CAF-SULF2, glypican-3 and tumour nuclear β-catenin, as well as cases reminiscent of in vitro data − with membranous accumulation of β-catenin − there were no consistent, significant associations with CAF-SULF2 (data not shown). The lack of an association between SULF2 and glypican-3 in the TGCA dataset (Spearman Correlation −0.09, *p* = 0.09) corroborated these data. We concluded that while CAF-SULF2 contributes to the regulation of Glypican-3/Wnt and β-catenin in HCC, supported by in vitro data, the in vivo heterogeneity was in keeping with additional mechanisms contributing to the poor prognosis attributed to CAF-SULF2 in our case series.

### SULF2 Induces Activation of a PDGFRβ/STAT3 Pathway in the Stromal Cells

To gain further mechanistic understanding of how CAF-SULF2 may modulate the TME, we next explored SULF2 regulated signalling in stromal cells themselves. An assessment of candidate fibroblast phosphoproteins (AKT, ERK1/2, p65, STAT3 JNK, PDGFRβ) identified upregulation of phospho-STAT3 (pSTAT3) in TGFβ1-stimulated LX-2 cells or LX-2 cells over-expressing SULF2, compared to vehicle-treated and empty vector-transfected LX-2 cells, respectively (online suppl. Fig. 5Ai, Aii). Conversely, STAT3 phosphorylation was attenuated in SULF2 KD COS-7 cells compared to control COS-7 cells (online suppl. Fig. 5iii). Phosphorylation and activation of STAT3 in myofibroblasts is downstream of platelet derived growth factor receptor β (PDGFRβ), an established and key regulator of the TME, linked to tumour growth [21, 22]. In the TCGA, SULF2 expression was positively associated with the expression of PDGFRβ (Spearman's Rho 0.58, *q* value = 2.53e−32) and STAT3 (Spearman's Rho 0.22, *q* value = 1.505e−5). In vitro, expression of PDGFRβ was induced in TGFβ1-stimulated LX-2 cells or LX-2 cells over-expressing SULF2 but diminished in SULF2 KD COS-7 cells (online suppl. Fig. 5Ai–Aiii).

### Stromal SULF2 Associates with an Altered Tumour-Immune Cell Environment

**Fig. 5:**
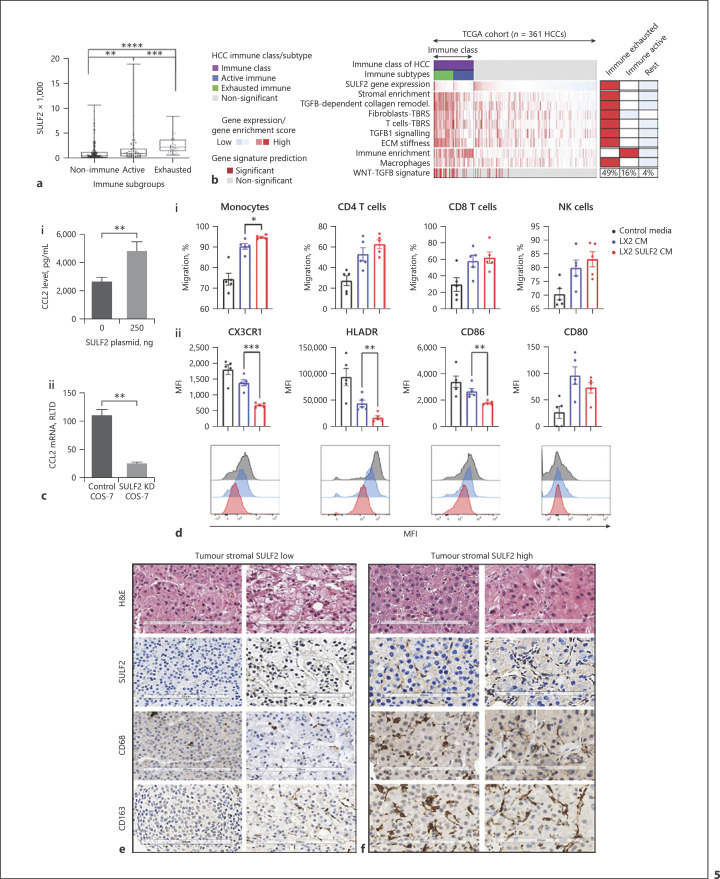
SULF2 expression characterizes an immune HCC phenotype, with stromal SULF2 upregulating CCL2 inducing, an altered macrophage phenotype and accumulation of macrophage in tumours. **a** SULF2 expression in HCC patients with an immune-exhausted phenotype was higher than patients with resting and immune active phenotypes (*p*< 0.001, independent sample Kruskal-Wallis test, n = 370 patients). **b** Heatmap of TCGA patients classified as Immune (exhausted and active) or nonimmune (rest) illustrates further how SULF2 expression correlates with TGFβ1 signalling, TGFβ1 regulated signatures (TBRS), and immune signatures. The median of the enrichment score in each class is shown on the right, with the percentage of patients in each class with enrichment of the WNT-TGFB signature. An ELISA assay confirmed SULF2 overexpression in LX-2 CM, with promoted secretion of CCL2 shown in (**ci**). **cii**CCL2 mRNA was suppressed in SULF2 KD COS-7 cells. **di**LX-2 SULF2-CM preferentially increased transwell migration of monocytes (macrophage precursors) relative to LX-2-CM. **dii**Characterization of CM activated CD80 expressing monocytes revealed an altered phenotype, with reduced expression of CX3CR1, HLADR, and CD86 in the presence of SULF2. Representative images of H&E, SULF2, CD68, and CD163 immunohistochemistry in patients with low (**e**) versus high (**f**) tumour stromal SULF2 expression showed higher numbers of CD68 or CD163 positive macrophages in tumours with high SULF2.

We went on to explore the impact of CAF-SULF on the TME. The previously defined HCC immune-exhausted subclass [23] is characterized by active tumour stroma, enrichment of the TGFβ1 pathway, and the poorest patient prognosis. Analysis of SULF2 mRNA expression levels in the TCGA dataset revealed that the *SULF2* level was significantly elevated in the immune compared to nonimmune phenotypes, particularly in the immune-exhausted phenotype (*p* < 0.000; independent-samples Kruskal-Wallis test, *n* = 372 patients) (Fig. 5a). SULF2 gene expression correlated significantly with the TGFβ1 collagen remodelling signature (*r* = 0.481, *p* < 0.001), as well as fibroblasts and T cells TGFβ response signatures (TBRS) signatures (Spearman *r* = 0.308 and *r* = 0.397, *p* < 0.001), important for metastasis initiation [24], which were all enriched in the immune-exhausted class, along with the WNT-TGFB signature [25] (Fig. 5b). In 20 patients with advanced disease (online suppl. Table 5), SULF2 positivity in CAFs was associated with CD45 positive immune infiltrates (Spearman correlation 0.688, *p* = 0.005), underpinned by associations with macrophage markers CD68 and CD163, rather than T lymphocytes (online suppl. Table 6). CCL2 is a macrophage chemoattractant released from activated myofibroblasts and CAFs [26]. In vitro overexpression of SULF2 in LX-2 cells significantly increased the protein level of CCL2, while SULF2 KD in COS-7 cells significantly reduced *CCL2* expression (Fig. 5c). In vitro, migration of human monocytes (macrophage precursors), isolated from peripheral blood mononuclear cells (PBMC) from healthy volunteers, was significantly enhanced in SULF2-CM compared to CM without SULF2, while that of lymphocytes was not (Fig. 5f). Furthermore, while SULF-CM had no impact on a panel of lymphocyte phenotypic markers, (online suppl. Fig. 5B) in SULF2-CM activated monocytes (upregulated CD80), expression of CX3CR1, CD86, and HLADR was suppressed (Fig. 5g). Reduced HLADR and CD86 are features of immunosuppressive monocytes in cancer [27, 28, 29], while loss of CX3CR1/CX3CL1 signalling in monocytes and TME can accelerate murine tumour growth [30]. Examples of CAF-SUL2 low versus CAF-SULF2 high tumours, with corresponding macrophage infiltration, are shown in Figure 5e.

### Paracrine Regulation of Tumour IKKβ/NF-κB Signalling Axis by Stromal SULF2

**Fig. 6:**
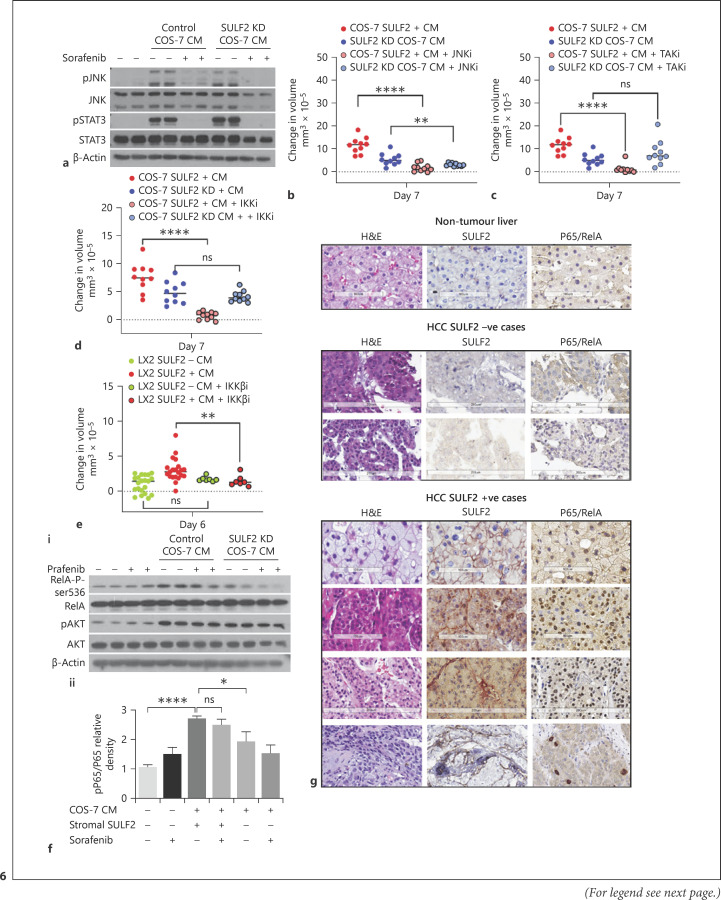
Stromal SULF2-dependent Hep3B growth and sorafenib sensitivity were regulated by TAK1/IKKβ/NF-κB RelA-P-ser536, with nuclear p65 associated with stromal SULF2. **a** Western blotting of Hep3B whole cell lysates, showed that activation of JNK/pJNK and STAT3/pSTAT3 pathways in Hep3B cells was driven by COS-7 CM independently of SULF2 and was sensitive to sorafenib treatment. **b** JNK inhibition limited Hep3B spheroid growth in both SULF2-dependent and independent fashion. In contrast, TAK1 (**c**) or IKKβ (**d**) inhibition limited SULF2-dependant Hep3B spheroid growth. **e** Similarly, Hep3B spheroid growth promoted by CM from SULF2-expressing LX-2 cells was suppressed by IKKβ inhibition. **fi**Activation of RelA-P-ser536 in Hep3B cells was dependent on control COS-7 CM and persistent in the presence of sorafenib, as was AKT activation. Quantification of RelA-P-ser536 (p-p65) relative to total p65 detected by Western blot is shown in **fii**, where data are mean ± SEM of 4 experiments. **g** IHC staining confirmed that SULF2 positivity in stromal cells associated with nuclear RelA-P-ser536 in adjacent tumour cells. Images are at ×20 magnification; scale bars represent 100–200 microns. Hep3B spheroid data are expressed as mean ± SEM of *n* = 10 experimental repeats. ns, not significant, *p = 0.05,**p < 0.01; ***p < 0.001, ****p< 0.0001.

We went on to explore the contribution of SULF2 to paracrine crosstalk in the TME, using inhibitors of inflammation associated JNK, TAK1, and IKKβ signalling pathways. Stromal CM induced activation of JNK and STAT3 pathways in the tumour cells in the presence and absence of SULF2 (Fig. 6a), with JNK inhibition suppressing both SULF2-dependent and independent Hep3B spheroids growth (Fig. 6b). Conversely, SULF2-dependent growth of Hep3B spheroids was suppressed in the presence of either TAK1 or IKKβ inhibitors (Fig. 6c-e). TAK1 and IKKβ are kinases upstream of NF-κB signalling, with subsequent Western blotting confirming stromal SULF2 from COS-7 cells induced phosphorylation of the downstream target, NF-κB subunit RelA at serine 536 (RelA-P-ser536) (Fig. 6f). In the Hep3B spheroids, SULF2-induced RelA-P-ser536 persisted when cells were treated with sorafenib in the presence of control COS-7 SULF2-containing CM, but not when sorafenib was added to SULF2 KD COS-7 CM (Fig. 6e). Sorafenib inhibition of JNK and STAT3 phosphorylation was similar regardless of CM containing SULF2. These in vitro data are in keeping with NF-κB activation contributing to stromal SULF2-induced sorafenib resistance.

In our patient series, nuclear localization of RelA-P-ser536 in tumour cells was a dramatic feature in HCC biopsies with high levels of SULF2 (Fig. 6g). Nuclear RelA-P-ser536 positivity was scant/absent in tumour cells in the absence of SULF2. Nuclear RelA-P-ser536 in tumour cells and the presence of CAF-SULF2 were strongly correlated (Spearman's rho 0.722; *p* < 0.0001, Pearson χ^2^ test 0.005), supporting stromal SULF2 as a regulator of a RelA-P-ser536 in human HCC in vivo.

## Discussion/Conclusion

CAFs are a major source for growth factors, cytokines, and morphogens, and they comprise a marker of poorer prognosis in the TME [31, 32]. Our study of histological SULF2 protein expression in biopsy rather than resection tissues has advanced data from previous transcriptomic datasets. In our cohort, SULF2 upregulation was in CAFs rather than in tumour cells in the majority of cases with a dramatic and detrimental impact on patient survival. SULF2 loss-of-function, gain-of-function, and SULF2 antibody studies were explored in 2D and 3D co-culture systems, confirming paracrine CAF-SULF2-induced proliferation, migration, invasion, and sorafenib resistance in HCC cells.

Mechanistically, we first explored the impact of exogenous SULF2 on glypican-3/Wnt signalling and while it is known that SULF2 modulates this pathway in cancers, the mutational status of β-catenin and other Wnt signalling aberrations were unknown in our biopsy cases and the additional contribution of SULF2 was unclear. SULF2 promotes Wnt/β–catenin signalling by 6-O desulfation of the morphogen Glyipcan-3 and is also reported to elevate glypican-3 expression. Our in vitro data showed that CM containing SULF2 promoted activation of a β–catenin reporter and increased Glypican-3 in HCC cells. In vivo, there was no clear-cut significant correlations between SULF2, glypican-3 and β-catenin immunohistochemistry. Given that there was a clear association between CAF-SULF2 and poor prognosis, we explored additional candidate mechanisms.

We considered TME signalling pathways in a wider context, showing that TGFβ-1-mediated upregulation of CAF-SULF2 activated PDGFβ/STAT3 pathway in CAFs, whilst also increasing the phosphorylation and activation of NF-κB pathway in the adjacent tumour cells, conferring therapy resistance. These findings are in keeping with growing insights into CLD and cancers. The PDGF/PDGFR signalling pathway is of established paramount importance in these fields [33, 34, 35, 36], with TGFβ1-mediated induction of metastasis and epithelial-mesenchymal transition also attributed to upregulation of stromal PDGFRβ [36]. STAT3, a downstream target of PDGFRβ in nonparenchymal cells [37], is also upregulated in the TGF-β1 rich profibrotic niche [38] and regarded as a hallmark of liver inflammation and malignancy [39]. Of note, both activation of STAT3 and NF-κB pathways in non-tumour liver tissues have established prognostic relevance in patients with HCC [39, 40]. The NF-κB pathway is also activated in many types of inflammation-associated cancers [41], often with advanced disease. It has also been associated with resistance or poor tolerability to chemotherapeutic agents in breast cancer [42], head and neck squamous cell carcinoma [43], and prostate cancer [44]. In HCC, in vitro data supporting a role for SULF2 as a mediator of sorafenib resistance has been previously reported, with SULF2 suppression or loss-of-function (SULF2N491K mutation) altering sorafenib sensitivity [45]. Our data add to this growing literature, highlighting the roles of stromal SULF2 and as an upstream regulator of TME TAK1/IKKβ/NF-κB signalling, contributing to therapy resistance. The direct mechanism remains to be elucidated. FGF/FGFR signalling, for example, is known to be modulated by SULF2 interaction with HS, with recent studies identifying FGFR1 as an upstream activator of TAK1 [46]. TGCA associations between SULF2 and FGFR1 were very highly significant (FGFR1 0.6281 *p* = 1.39–39).

Perhaps the most compelling aspect of this study, given the current excitement centred on the role of immuno-oncology therapies in HCC and the need to understand what underpins response to treatments, is the identified SULF2 association with the HCC immune class [23] and the possibility that SULF2 has a role to play in immune exhaustion. Evident in around 25% of HCC patients, the HCC immune subclass is divided into an active immune subclass and a TGFβ1 exhausted immune subclass, with the enrichment of *SULF2* in this latter group is noteworthy. Rather than simply a bystander observation, our data support a role for SULF2 in the recruitment of tumour-associated macrophage into the cancer niche, via release of the chemoattractant CCL2. In pancreatic and colorectal cancers, αSMA positive CAFs recruit tumour-associated macrophages via secretion of IL6, CCL2, G-CSF, TGFβ1, and FAP [47, 48], while activation of PDGFRβ/STAT3 is associated with CCL2 release [49, 50]. Here we have shown stromal SULF2 release following TGFβ1 stimulation, a striking correlation in vivo between SULF2 and TGFβ1, as well as TGFβ1 regulated TME signatures, SULF2 activation of stromal PDGFRβ/STAT3 and release of CCL2, with a significant association between stromal SULF2 and tumour macrophages in vivo. In vitro studies on human PBMCs have confirmed that the presence of SULF2 preferentially induces monocyte migration and induces in them a phenotype associated with immunosuppression and accelerated tumour growth.

In conclusion, we have provided tissue evidence and in vitro mechanistic data, supporting SULF2 as a potentially critical mediator of TME-driven HCC progression − worthy of further investigation as a prognostic and predictive biomarker but also as a therapeutic target alone or in combination with other therapies.

## Statement of Ethics

The use of patients tissues surplus to diagnostic requirements for research purposes was approved by the Newcastle and North Tyneside Regional Ethics Committee (REC), the Newcastle Academic Health Partners Bioresource (NAHPB), and the Newcastle upon Tyne NHS Foundation Trust Research and Development (R&D) department, in accordance with Health Research Authority guidelines (References REC 2004/012; REC 04/Q0905/168; REC 10/H0906/41; NAHPB Project 48; REC 12/NE/0395; R&D 6579; REC 19/NE/0251; R&D 8683; Human Tissue Act license 12534). Patients were over 18 years of age and the majority provided written informed consent. Initial studies including optimization of antibodies and characterization of SULF2 in patient tissues included a small number of retrospective cases with tissue blocks in the pathology department (*n* = 6, 2000–2002). These were from deceased patients for whom the REC waived consent.

## Conflict of Interest Statement

F.O. is a director and shareholder in Fibrofind Ltd. J.L. is a shareholder in Fibrofind Ltd, all other authors declare no competing interests.

## Funding Sources

M.Y.W.Z. was supported by a personal award from the Newton-Mosharafa Fund and is currently funded by Newton prize 2020; S.F.A. was supported by a personal award from Damascus University; G.L.P. and the creation of the Newcastle University Gastroenterology Research Tissue Bank were supported by the European Community's Seventh Framework Program (FP7/2001–2013) under Grant agreement HEALTH-F2-2009-241762 for the project FLIP. H.L.R, D.T., A.W., H.T., G.S.B. were supported by Newcastle Cancer Research UK (CRUK) Experimental Cancer Medicine Centre award C9380/A18084. H.L.R., F.O., J.L. and M.L. were supported by the CRUK Accelerator award C9380/A26813 and CRUK programme Grant C18342/A23390. H.L.R., F.O., Q.M.A., M.Y.W.Z. and O.G. were supported by Horizon 2020 Framework Program of the European Union under Grant Agreement 634413 for the project EPOS. F.O. received Medical Research Council funding, program Grants MR/K0019494/1 and MR/R023026/1. D.G. is funded by the Newcastle CRUK Clinical Academic Training Programme.

## Author Contributions

Marco Y.W. Zaki and Sari F. Alhasan − acquisition of data analysis and interpretation of data; drafting of the manuscript; critical revision of the manuscript for important intellectual content; statistical analysis; and obtained funding. Ruchi Shukla − acquisition of data; analysis and interpretation of data; drafting of the manuscript; critical revision of the manuscript for important intellectual content; statistical analysis; and study supervision. Misti McCain, Gillian L. Patman, Despina Televantou, Lee A. Borthwick, and Olivier Govaere − acquisition of data; analysis and interpretation of data; critical revision of the manuscript for important intellectual content; and study supervision. Maja Laszczewska, Daniel Geh, and Daniela Sia − acquisition of data; analysis and interpretation of data; critical revision of the manuscript for important intellectual content; and statistical analysis. Anna Whitehead − acquisition of data; analysis and interpretation of data; and critical revision of the originally submitted manuscript for important intellectual content. João P. Maurício, Ben Barksby, Lucy M. Gee, and Hannah L. Paish − acquisition of data; analysis and interpretation of data; and critical revision of the manuscript for important intellectual content. Jack Leslie, Ramy Younes, Huw Thomas, and Gary S. Beale − analysis and interpretation of data; critical revision of the manuscript for important intellectual content; and study supervision. Alastair D. Burt − study concept and design; analysis and interpretation of data; critical revision of the manuscript for important intellectual content; and study supervision. Quentin M. Anstee − analysis and interpretation of data; critical revision of the manuscript for important intellectual content; obtained funding; and study supervision. Dina Tiniakos − acquisition of data; analysis and interpretation of data; drafting of the manuscript; critical revision of the manuscript for important intellectual content; and study supervision. Fiona Oakley − study concept and design; acquisition of data; analysis and interpretation of data; drafting of the manuscript; critical revision of the manuscript for important intellectual content; statistical analysis; obtained funding; study supervision; and study supervision. Helen L. Reeves − study concept and design; acquisition of data; analysis and interpretation of data; drafting of the manuscript; critical revision of the manuscript for important intellectual content; statistical analysis; obtained funding; and study supervision.

## Data Availability Statement

All data generated or analysed during this study are included in this article and its online supplementary material. Further enquiries can be directed to the corresponding author.

## Supplementary Material

Supplementary data

## Figures and Tables

**Table 1:** Demographic and clinicopathological features of patients

	All patients	SULF2 in HCC cells			SULF2 in CAFs		
	60	absent 51	present 9	*p* value	absent 29	present 31	*p* value
Age (median)	69	69	65	ns	69	69	ns
Gender (male/female)	49/11	42/9	7/2	ns	23/6	26/5	ns
BMI (median)	27	27	27	ns	25	28	ns
T2DM no/yes	32/28	30/21	2/7	0.042*	14/15	18/13	ns
Cirrhosis no/yes	31/29	25/26	6/3	ns	14/15	17/14	ns
CLD none/ALD/NAFLD/other	19/10/15/16	16/9/13/13	3/1/2/3	ns	9/4/7/9	10/6/8/7	ns
Grade 1/2/3	18/27/15	17/23/11	1/4/4	ns	12/10/7	6/17/8	ns
Size (cm)	6.7±0.7	6.2±0.8	9.6±1.6	0.026*	6.2±1.1	7.2±1.0	ns
Tumour number	1.7±0.1	1.6±0.1	2.3±0.5	0.11	1.6±0.2	1.9±0.2	ns
PVT no/yes	52/8	46/6	6/3	0.056	27/2	25/6	ns
EHD no/yes	52/8	47/4	5/4	0.003**	27/2	25/6	ns
TNM stage 1/2/3/4	29/12/11/8	26/11/10/4	3/1/1/4	0.034*	12/7/3/2	15/2/8/6	ns
INR	1.0±0.02	1.0±0.02	1.0±0.03	ns	1.0±0.02	1.03±0.04	ns
Albumin (g/L)	38.8±0.67	39.2±0.7	36.8±1.9	ns	39.4±0.9	38.3±1.0	ns
Bilirubin (µmol/L)	15.1±1.9	14.1±1.1	21.2±10	ns	12±0.9	18.1±3.4	ns
AFP (median)	6	5	1,400	0.03*	6	6	ns
Ascites no/yes	55/5	47/4	8/1	ns	28/1	27/4	ns
Childs-Pugh A/B/C	53/6/1	46/5/0	7/1/1	ns	28/1/0	25/5/1	ns
BCLC stage A/B/C/D	17/13/28/2	15/12/22/2	2/1/6/0	ns	10/7/12/0	7/6/16/2	ns
ECOG PST 0/1/2	32/22/6	28/19/4	4/3/2	ns	17/10/2	15/12/4	ns
Therapy OLTx/Res/ablation	3/7/12	3/5/12	0/2/0	0.037*	2/2/8/	1/5/4	ns
TACE/Med/BSC	28/1/9	25/0/6	3/1/3		14/1/2	14/1/7	
Median survival (months)	20.3	28.7	11.6	ns	35.0	12.2	ns
No surgical treatment	*n* = 50	*n* = 43	*n* = 7		*n* = 25	*n* = 25	
Median survival (months)	16.8	19.7	9.9	0.060	29.2	7.2	0.003**

TNM, tumour node metastases; PST, performance status. Continuous data are presented as mean±standard error unless otherwise stated, with statistical comparisons using a Mann-Whitney test. Categorical data were compared using a χ^2^ test. Survival was assessed by the Kaplan-Meier method. “Other” included small numbers with Hepatitis C (*n* = 4); haemochromatosis (*n* = 4), cryptogenic cirrhosis (*n* = 4); Hepatitis B (*n* = 1); autoimmune hepatitis (*n* = 2); and α-1-antitrypsin deficiency (*n* = 1).

**Table 2:** Multivariate analysis of factors associated with survival in nonsurgically treated patients

Variable	UVA	MVA entering elevated stromal SULF2	
	univariate *p* value	multivariate *p* value	HR (CI)
Age	0.258		
Gender	0.195		
AFP	0.014	0.019	1.0 (1.0–1.0)
Tumour number	0.195		
Tumour size	0.012	0.278	1.1 (0.96–1.17)
EHD	0.006	0.740	
PVT	<0.001	0.146	
Edmondson-Steiner grade			
Grade 1 (*n* = 17)	0.006	0.114	
Grade 2 (*n* = 22)	0.001	0.069	
Grade 3 (*n* = 11)	0.014	0.553	
Cirrhosis	0.976		
Ascites	<0.001	0.292	
Albumin	<0.001	0.001	0.87 (0.79–0.94)
Bilirubin	0.227		
INR	0.681		
ECOG PST			
PST 0 (*n* = 23)	<0.001	0.026	
PST 1 (*n* = 21)	<0.001	0.693	0.63 (0.07–6.10)
PST 2 (*n* = 6)	0.01	0.416	2.57 (0.26–24.9)
Tumour SULF2			
Absent (*n* = 43)	0.061		
Present (*n* = 7)			
Stromal SULF2	0.006	0.035	0.44 (0.20–0.94)
Absent (*n* = 25) Present (*n* = 25)			

PST, performance status; HR, hazards ratio. Factors associated with survival in 50 patients for whom surgical treatment was not an option were assessed by univariate analysis. Factors with a *p* value <0.01 were entered into a multivariate Cox regression analysis. Two distinct multivariate analyses are shown, the first considering stromal SULF2 and the second (shaded in grey) showing a similar analysis, but in which SULF2 overexpression in either HCC cells or stromal cells was classed as “present.” Significance and HR with upper and lower 95% confidence intervals are shown.
