# Supplementary material for: Sulfatase-2 from Cancer Associated Fibroblasts: An Environmental Target for Hepatocellular Carcinoma?
Source: Liver Cancer. 2022 Jul 13;11(6):540–57. doi: 10.1159/000525375 (PMC9801184; doi:10.1159/000525375)
Supplement: Supplementary file 1 — Supplementary data [file lic-0011-0540-s01.docx]

# Sulfatase-2 from cancer associated fibroblasts – an environmental target for hepatocellular carcinoma?

Marco Y. W. Zaki, Sari F. Alhasan, Ruchi Shukla, Misti McCain, Maja Laszczewsk, Daniel Geh, Gillian L. Patman, Despina Televantou, Anna Whitehead, João P. Maurício, Ben Barksby, Lucy M. Gee, Hannah L. Paish, Jack Leslie, Ramy Younes, Alastair D. Burt, Lee A. Borthwick, Huw Thomas, Gary S. Beale, Olivier Govaere, Daniela Sia, Quentin M. Anstee, Dina Tiniakos, Fiona Oakley, Helen L. Reeves.

Index

[Supplementary Materials and Methods 2](#_Toc77745054)

[Supplementary Tables 6](#_Toc77745055)

[Supplementary Results Figures 10](#_Toc77745056)

[Supplementary references 15](#_Toc77745057)

## Supplementary Materials and Methods

Cell culture

Stromal cells grown in 10% FBS conditioned media (CM) was collected after 48 hours, while CM from stromal cells in serum-free media was collected 24 hours and concentrated using Amicon® Ultra-15 tubes (Merk Millipore, UK, nr UFC900324). The concentration of SULF2 in the CM was measured using Human SULF2 ELISA kit (LifeSpanBioSciences, Inc, WA, nr LS-F12678-1) according to the manufacturer protocol. Sorafenib was purchased from Cell signalling technologies (nr 8705). SP600125 (JUN N-Terminal Kinase (JNK) inhibitor, nr 1496), (5Z)-7-Oxozeaenol (transforming growth factor-β-activated kinase 1 (TAK1) inhibitor, nr 3604) and FR180204 (Selective Extracellular Signal- Regulated Kinase1/2 (ERK1/2) inhibitor, nr 3706) pathway inhibitors were purchased from Tocris. IKK-2 Inhibitor IV (IκB Kinase-Beta, nr 401483) was purchased from Calbiochem.

Isolation of Cancer associated fibroblasts (CAFs)

Human HCC patient biopsy or resection tissue was digested using human tumour dissociation enzymes (Miltenyi Biotec, nr 130-095-929) at 37°C for 60-90 minutes in a shaking incubator at 170rpm. Digestion was stopped using cold wash media (DMEM high glucose media supplemented with 1% FBS, 1% penicillin and streptomycin and 1% Glutamine and Pyruvate), the cell suspension was centrifuged at 1000rpm for 5 min. The cell pellet was then washed with cold PBS and cells centrifuged at 800 rpm for 5 min. The pellet was washed with cold PBS and cells centrifuged at 600 rpm for 5 min. Cells were then resuspended in media, plated onto collagen (50μg/mL) coated plates and cultured in RPMI media supplemented with 10% FBS, 1% penicillin and streptomycin, 1% Glutamine and Pyruvate, 1x ITS, 40 ng/mL EGF, 10 μM ROCK inhibitor (Y-27632) (Sigma, nr SCM075) and 5μM TGFβ inhibitor (A83-01) (Tocris, nr 2939). Primary culture CAFs were isolated from mixed HCC-CAFs populations using differential trypsinisation (0.05%) and cultured on plastic in DMEM high glucose media supplemented with 16% FBS, 1% penicillin and streptomycin and 1% Glutamine and Pyruvate. Media was changed every 3-4 days, spun at maximum speed to clear, sterile filtered and snap frozen for subsequent Hep3B spheroid experiments.

Low adhesion plate spheroid cultures

3000 Hep3B cells (per well) were seeded in an agarose coated 96 well plates and cultured in 50μl DMEM high glucose media supplemented with 10% FBS, 1% penicillin and streptomycin and 1% Glutamine and Pyruvate. Spheroids were allowed to form over 48h, the media was then changed with a 1:4 ratio of serum-free culture media : CAF CM and then treated with either 0.5μl SULF2 antibody (AbDSerotec, MCA5692GA) or 0.5μl control IgG antibody (Abcam, ab170190). Brightfield images were taken daily at 10x magnification to monitor spheroid growth.

2D Cell proliferation assays

Huh7 and Hep3B cells were co-cultured in 24-well plates with control or SULF2 KD COS-7 cells grown on 3.0µm ThinCerts™-TC inserts (Greiner bio-one, Switzerland, nr 662631). MTT (Sigma-Aldrich, USA, nr M2128) or BrdU (Roche, Germany, nr 11647229001) assays were performed according to manufacturer protocol. Experiments were performed at least three times.

Cell migration and invasion assays

Tumour cells were cultured overnight in culture-inserts (Ibidi, Germany, nr 80209) then treated with stromal CM for 24 hours. Cell migration was measured and presented as %Gap closure. For cell invasion, control or SULF2 KD COS-7 cells were cultured overnight in 24-well plate. Boyden invasion chambers (Millipore, UK, nr ECM550) including Hep3B cells in serum-free media were then co-cultured with the stromal cells for 72 hours. Invaded tumour cells were quantified according to the manufacturer protocol.

Western blotting

Gel electrophoreses was performed using NuPAGE 4-12% Bis-tris gel and NuPAGE MOPS-SDS running buffer (Life Technologies, UK), proteins were then transferred to Nitrocellulose membrane (HyBond-C Extra, Amersham Biosciences, UK). List of antibodies are in **supplementary table 2**.

Immunofluorescence (IF) Cell Lines

Cells treated with stromal CM were fixed, blocked with 5% goat serum and treated with anti-β-catenin or anti-GPC3 primary antibodies for 1 hour, then incubated with secondary antibody for another hour. List of antibodies are in **supplementary table 2**. Cells were stained with DAPI and mounted according to manufacturer protocol.

Immunofluorescence (IF) Human cancer activated fibroblasts (CAFs)

CAFs were fixed with 4% paraformaldehyde for 10 mins at room temperature. CAF cell membranes were permeabilized using 0.1% Triton X-100 and non-specific binding was blocked using blocking buffer (1% Bovine Serum Albumin in PBS). CAFs were stained overnight at 4^o^C with αSMA-FITC conjugated (Sigma clone 1a4, 1:250) and SULF2 primary (AbDSerotec, MCA5692GA, 1:200) antibodies diluted in blocking buffer. The next day the CAFs were washed three times in PBS, then incubated with AF594 conjugated donkey anti-rabbit secondary antibody at 1:1000 (Invitrogen, nr A32754). Nuclei were stained using Hoechst solution, prior to mounting with ProLong™ Glass Antifade Mountant (Invitrogen™, nr P36984). Cells were imaged using Leica SP8 STED confocal microscope.

RNA extraction and quantitative RT-PCR

RNA from CAFs was extracted using the RNeasy Micro Kit (Qiagen), whilst RNA from all other cell lines using RNeasy PlusMini Kit (Qiagen) according to the manufacturers’ instructions. Eluted RNA was treated with DNAse followed by reversetranscription into complementary DNA (cDNA) using iScript cDNA synthesis kit (BioRad, nr 1708891) according to the manufacturer’s protocol. Designed sequences of the primers for genes of interest are listed in **supplementary table3**.

CCL2 ELISA

CAF-CCL2 was measured DuoSet ELISA (R&D Systems, DY279) according to the manufacturer’s protocol. Briefly, CCL2 capture antibodies, diluted in PBS, were added to 96 well plates (DY990), sealed and incubated overnight with rocking. Plates were blocked using DY995 reagent diluent (RD, 1% BSA + PBS) for 1 hour at RT. Recombinant protein was diluted (6 step 2-fold serial dilution) using RD to a concentration of 2000 pg/ml for the standard curve. Samples were diluted in RD to an assay dependant concentration. The plate was washed 3 times with PBS + 0.05% Tween (WA126) then samples and standards were incubated in the plate with rocking at RT for 2 hours. After washing, the detection antibodies were added and incubated for 2 hours at RT with rocking. Streptavidin conjugated to horseradish protein was added to the plate and then incubated in the dark for 20 minutes. Plates were washed prior to application of substrate solution (1:1 ratio) (DY999). The reaction was terminated by adding stop solution (1M H_2_SO_4_). The optical density was measured at 595nm and 450nm.

SULF2 ELISA

SULF2 levels in control and SULF2 KD COS-7 cells were performed using Human SULF2/Sulfatase 2 (Sandwich ELISA) ELISA Kit (LS-F12678) according to the manufacturer’s protocol. Briefly, recombinant SULF2 protein was reconstituted in 2 ml sample diluent and diluted (7 step 2-fold serial dilution) to a concentration of 313 pg/ml for the standard curve. Samples and standard solutions were pipetted to each well of 96-well plate and incubated for 2 hours at 37◦C. After aspiration of samples/standards, the detection reagent was added and incubated for 1 hour at 37◦C. The reagent was aspirated, wells were washed three times and detection reagent 2 was added and incubated for 1 hour at 37◦C. All reagents were then aspirated and wells were washed 5 times before adding the TMB substrate for 15 minutes at 37◦C sealed with aluminium foil. Stop solution was added and the optical density (OD) was measured at 450 nm.

Peripheral Blood Mononuclear Cells (PBMC) isolation

Informed consent was obtained from healthy volunteers, with institutional ethical approval, and 40ml of blood collected into heparin coated tubes. Peripheral blood mononuclear cells (PBMCs) were subsequently isolated using a Ficoll gradient and counted before being used for the transwell migration assay and media culture experiment.

Transwell Migration Assay

PBMCs were resuspended in RPMI media without FBS and added to the inserts of a 5 μm transwell migration assay plate (Corning, CLS3421) at 1.5 million PBMCs per well. Complete RPMI media (control), conditioned media from Lx2 cells transfected with pcdna vector (normal SULF2 expression) and conditioned media from Lx2 cells transfected with SULF2 vector (SULF2 overexpression) were added to the bottom wells. The assay plate was then incubated at 37°C in an atmosphere of 5% CO_2_ for 48 hours. PBMCs from both the insert and bottom well were then collected for flow cytometry analysis.

PBMC in culture media experiment

PBMCs were resuspended in either complete RPMI media (control), conditioned media from Lx2 cells transfected with pcdna3 vector (normal SULF2 expression) and conditioned media from Lx2 cells transfected with SULF2 vector (SULF2 overexpression) and plated into a 96 well culture plate at 1.5 million cells per well. Cells were then incubated at 37°C in an atmosphere of 5% CO_2_ for 48 hours before flow cytometry analysis.

Flow Cytometry

Dead cells were stained with cell viability stain (ThermoFisher, LIVE/DEAD fixable blue stain) followed by Fc blockade (Biolegend, Human TruStain FcX). Cells were then stained with a panel of previously validated antibodies (**supplementary table 2**). Samples were run on the BD FACSymphony cytometer with optimised voltages and compensation set. Flow cytometry analysis was done using FlowJo v9. Samples were gated as shown below for monocytes, CD4 T cells, CD8 T cells and NK cells. Medium fluorescence intensities (MFIs) of activation and phenotypic markers were then compared between samples. For the transwell migration assay percentage of each cell type migrating across the membrane was calculated by dividing the number of cells in the bottom well by the total number of cells in both the bottom well and insert.


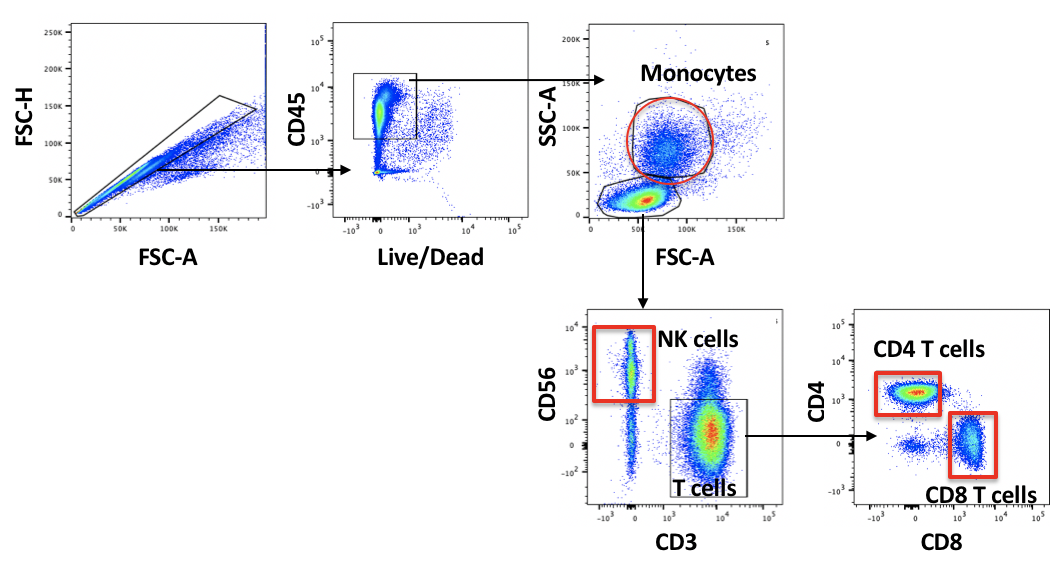


## Supplementary Tables

Supplementary table 1: shRNA sequences of mission TRC2 SULF2 and non-targeting shRNA lentiviral particles.

| **Target** | **Sequence** |
| --- | --- |
| SULF2 shRNA | CCGGGGGCGAAAGTCATTGGAATTTCTCGAGAAATTCCAATGACTTTCGCCCTTTTTG |
| NT shRNA | CCGGCAACAAGATGAAGAGCACCAACTCGAGTTGGTGCTCTTCATCTTGTTGTTTTT |

Supplementary table 2: List of antibodies:

| **Staining** | **Catalogue number** | **Producer** | **Clonality/host species** | | **Antigen retrieval (IHC)** | **Experiment** |
| --- | --- | --- | --- | --- | --- | --- |
| SULF2 | MCA5692GA | AbDSerotec | Mouse mAb | | Triology, Cell Marque,UK | IHC (1:200),  WB (1:1000) |
| αSMA | ab7817 | Abcam | Mouse mAb | | Pressure cooker | IHC (1:200) |
| Phospho- NF-Kb p65 (Ser536) | ab86299 | Abcam | Rabbit pAb | | Tris buffer  (pH 9) | IHC(1:200) |
| CD45 | Ab10558 | Abcam | Rabbit pAb | | Ventana Citrate | IHC(1:200) |
| CD4 | 790-4423 | Roche | RabbitmAb  (clone SP35) | | Ventana Citrate | Ventana standard protocol |
| CD8 | 790-4460 | Roche | Rabbit mAb  (clone SP57) | | Ventana Citrate | Ventana standard protocol |
| CD66b | 305102 | BioLegend | Mouse mAb | | Ventana Citrate | IHC (1:600) |
| CD68 | 790-2931 | Roche | Mouse mAb  (Clone KP1) | | Ventana Citrate | Ventana standard protocol |
| Isotype control | ab170190 | Abcam | Mouse mAb | |  | *In vitro* |
| Phosphor-SAPK/JNK (Thr183/Tyr185) | 9251 | Cell signalling | Rabbit pAb | |  | WB (1:1000) |
| SAPK/JNK (Thr183/Tyr185) | 9252 | Cell signalling | Rabbit pAb | |  | WB (1:1000) |
| Phospho- NF-Kb p65 (Ser536)(93H1) | 3033 | Cell signalling | Rabbit mAb | |  | WB (1:1000) |
| NF-Kb p65 (C-20) | sc-372 | Santa cruz biotechnology | Rabbit pAb | |  | WB (1:1000) |
| Phospho-stat3 (Tyr705)(D3A7) | 9145 | Cell signalling | Rabbit pAb | |  | WB(1:1000) |
| Stat3 | 9132 | Cell signalling | Rabbit pAb | |  | WB(1:1000) |
| Phospho-p44/42 MAPK (ERK1/2) (Thr202/Tyr204) | 9101 | Cell signalling | Rabbit pAb | |  | WB(1:1000) |
| p44/42 MAPK (ERK1/2) | 0102 | Cell signalling | Rabbit pAb | |  | WB(1:1000) |
| phospho-AKT | 4060 | Cell signalling | RabbitmAb | |  | WB (1:2000) |
| AKT | 4691 | Cell signalling | Rabbit mAb | |  | WB (1:2000) |
| GAPDH | ab37168 | Abcam | RabbitpAb | |  | WB (1:2000) |
| B-actin | A-5441 | Sigma-Aldrich | Mouse mAb | |  | WB(1:2000) |
| CD45 (FITC) | 268508 | Biolegend | | Mouse mAb |  | FC (1:100) |
| CD8 (PerCP-Cy5.5) | 344709 | Biolegend | | Mouse mAb |  | FC (1:100) |
| CD69 (PE-Dazzle 594) | 310941 | Biolegend | | Mouse mAb |  | FC (1:100) |
| CD11c (PE-Cy5) | 561692 | Biolegend | | Mouse mAb |  | FC (1:100) |
| CD244 (PE-Cy7) | 329519 | Biolegend | | Mouse mAb |  | FC (1:100) |
| CCR2 (APC) | 357207 | Biolegend | | Mouse mAb |  | FC (1:100) |
| CD4 (AlexFluor 700) | 357417 | Biolegend | | Mouse mAb |  | FC (1:100) |
| CX3CR1 (APC/Fire 750) | 341631 | Biolegend | | Mouse mAb |  | FC (1:100) |
| CD3 (BV 421) | 300433 | Biolegend | | Mouse mAb |  | FC (1:100) |
| CD16 (BV510) | 360729 | Biolegend | | Mouse mAb |  | FC (1:100) |
| CD36 (BV605) | 563518 | BD Biosciences | | Mouse mAb |  | FC (1:100) |
| CD86 (BV650) | 305427 | Biolegend | | Mouse mAb |  | FC (1:100) |
| HLADR (BV711) | 563696 | BD Biosciences | | Mouse mAb |  | FC (1:100) |
| CD80 (BV785) | 305237 | Biolegend | | Mouse mAb |  | FC (1:100) |
| PD1 (BV750) | 329965 | Biolegend | | Mouse mAb |  | FC (1:100) |
| CD14 (BUV395) | 740286 | BD Biosciences | | Mouse mAb |  | FC (1:100) |
| CD25 (BUV563) | 741365 | BD Biosciences | | Mouse mAb |  | FC (1:100) |
| CD56 (BUV737) | 741842 | BD Biosciences | | Mouse mAb |  | FC (1:100) |
| CD62L (BUV805) | 741843 | BD Biosciences | | Mouse mAb |  | FC (1:100) |

Supplementary table 3: Primer information for Real time PCR

| **Gene** | **Forward primer 5’-3’** | **Reverse primer 5’-3’** |
| --- | --- | --- |
| SULF2 | ATGAGTTTGACATCAGGGTCCCGT | ATGGATTTCCCGTCCATATCCGCA |
| CCL2 | TGTCCCAAAGAAGCTGTGGAT | CTGCAGATTCTTGGGTTGTH |
| aSMA | GCGTGGCTATTCCTTCGTTACT | CCGATGAAGGATGGCTGGAACA |
| HPRT | TTGCTTTCCTTGGTCAGGCA | ATCCAACACTTCGTGGGGTC |
| GAPDH | CAATGACCCCTTCATTGACC | GATCTCGCTCCTGGAAGATG |

| **Experiment – Change in Spheroid volume** | **Change in volume** | **p-value** |
| --- | --- | --- |
| **CM from control versus TGF-β1-stimulated LX-2 cells** | | |
| Hep3B spheroids in TGF-β1 stim. LX-2 CM | 1.25 ± 0.204 | 0.0014 |
| Hep3B spheroids in control LX-2 CM | 0.64 ± 0.095 |  |
| **CM from empty vector versus SULF2 overexpressing LX-2 cells** | | |
| Hep3B spheroids in CM from LX-2 cells overexpressing SULF2 | 0.91 ± 0.276 | 0.0076 |
| Hep3B spheroids in CM from LX-2 cells transfected with empty vector | 0.38 ± 0.076 |  |
| **CM from control versus SULF2 KD COS-7 cells** | | |
| Hep3B in Hep3B CM | 0.81 ± 0.276 |  |
| Hep3B in SULF2 KD COS-7 CM | 1.43 ± 0.312 | <0.001 |
| Hep3B in control COS-7 CM | 2.68 ± 0.430 |  |
| **CM from TGF-β1-stimulated LX-2 cells** | | |
| Hep3B spheroids in TGF-β1 stim. LX-2 CM+ SULF2 Ab | 1.114 ± 0.116 | <0.0001 |
| Hep3B spheroids in TGF-β1 stim. LX-2 CM+ IgG | 1.643 ± 0.071 |  |
| **CM from control COS-7 cells** | | |
| Hep3B spheroids in control COS-7 CM +SULF2 Ab | 0.199 ± 0.053 | <0.0001 |
| Hep3B spheroids in control COS-7 CM + IgG | 0.8528 ± 0.16 |  |
| **CM from control versus TGF-β1-stimulated LX-2 cells** | | |
| Hep3B spheroids in TGF-β1 stim. LX-2 CM + 1.25μM Sorafenib | 0.269 ± 0.063 | <0.0001 |
| Hep3B spheroids in control LX-2 CM+ 1.25μM Sorafenib | -0.697 ± 0.079 |  |
| Hep3B spheroids in TGF-β1 stim. LX-2 CM + 1.25μM Sorafenib + SULF2 Ab | -0.42 ± 0.058 | <0.0001 |
| Hep3B spheroids in TGF-β1 stim. LX-2 CM+ 1.25μM Sorafenib + IgG | 0.239 ± 0.129 |  |
| **CM from control versus SULF2 KD COS-7 cells** | | |
| Hep3B in control COS-7 CM + Sorafenib | 3.198 ± 0.322 | <0.0001 |
| Hep3B in SULF2 KD COS-7 CM + Sorafenib | 0.75 ± 0.153 |  |
| Hep3B in control COS-7 CM + IgG control + Sorafenib | 3.155 ± 0.253 | <0.0001 |
| Hep3B in control COS-7CM + SULF2 mAb + Sorafenib | 1.989 ± 0.217 |  |
| **CM from control versus TGF-β1-stimulated LX-2 cells** | | |
| Hep3B spheroids in TGF-β1 stim. LX-2 CM | 0.96 ± 0.114 | 0.0012 |
| Hep3B spheroids in TGF-β1 stim. LX-2 CM + IKKβinh. | 0.47 ± 0.095 |  |
| Hep3B spheroids in control LX-2 CM | 0.322 ± 0.085 | 0.39 |
| Hep3B spheroids in control LX-2 CM+ IKKβinh. | 0.55 ± 0.041 |  |
| **CM from control versus SULF2 KD COS-7 cells** | | |
| Hep3B in control COS-7 CM | 2.41 ± 0.257 | <0.0001 |
| Hep3B in control COS-7 CM+ IKKβinh. | 0.23 ± 0.067 |  |
| Hep3B in SULF2 KD COS-7 CM | 1.53 ± 0.195 | 0.86 |
| Hep3B in SULF2 KD COS-7 CM + IKKβinh. | 1.34 ± 0.106 |  |

Supplementary table 4: change in spheroids volume in mm^3^±SEM for all spheroids experiments.

Supplementary Table 5. Independent cohort of patients referred for treatment with sorafenib

|  | **All patients** | **SULF2 in cancer associated fibroblasts (CAFs)** | | | | |
| --- | --- | --- | --- | --- | --- | --- |
|  | 20 | Absent/scant  10 | Present  10 | | p value |  |
| **Age** (years, median) | 72.5 | 72 | | 72.5 | ns |  |
| **Gender**(male/female) | 18/2 | 8/2 | | 10/0 | ns |  |
| **BMI** (median) | 28.65 | 28.5 | | 28.7 | ns |  |
| **T2DM** no/yes | 11/9 | 5/5 | | 6/4 | ns |  |
| **Cirrhosis** no/yes | 10/10 | 4/6 | | 6/4 | ns |  |
| **CLD** none/ALD/NAFLD/other | 4/4/8/4 | 2/1/3/4 | | 2/3/5/0 | ns |  |
| **Differentiation** well/mod/poor | 2/12/6 | 1/7/2 | | 1/5/4 | ns |  |
| **Size** (cm)(median) | 6.2 | 9.0 | | 4.5 | 0.02 |  |
| **Tumour number** | 3.0 | 2 | | 3 | ns |  |
| **PVT** no/yes | 17/3 | 9/1 | | 8/2 | ns |  |
| **EHD** no/yes | 18/2 | 9/1 | | 9/1 | ns |  |
| **TNM** stage 1/2/3/4 | 3/5/9/3 | 1/2/6/1 | | 2/3/3/2 | ns |  |
| **Childs-Pugh** A/B/C | 18/1/1 | 10/0/0 | | 8/1/1 | ns |  |
| **BCLC stage** A/B/C/D | 2/9/8/1 | 1/7/2/0 | | 1/2/6/1 | ns |  |
| **ECOG PST** 0/1/2 | 14/6 | 8/2 | | 6/4 | ns |  |
| **Sorafenib** yes/no | 18/2 | 9/1 | | 9/1 | ns |  |

Supplementary Table 6. Correlations between immunohistochemistry cell counts and the expression of CAF SUL2

|  |  | **CD45** | **CD4** | **CD8** | **CD66b** | **CD68** |
| --- | --- | --- | --- | --- | --- | --- |
| **CAF SULF2**  **correlation** | **Spearman** | 0.688 | 0.168 | 0.302 | 0.440 | 0.580 |
|  | **p value** | 0.005 | 0.533 | 0.274 | 0.176 | 0.019 |
|  | **number** | 15 | 16 | 15 | 11 | 16 |

## Supplementary Results Figures

Supplementary figure 1

**A B**


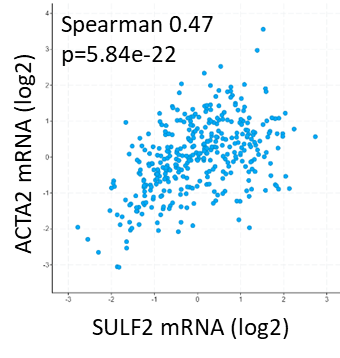

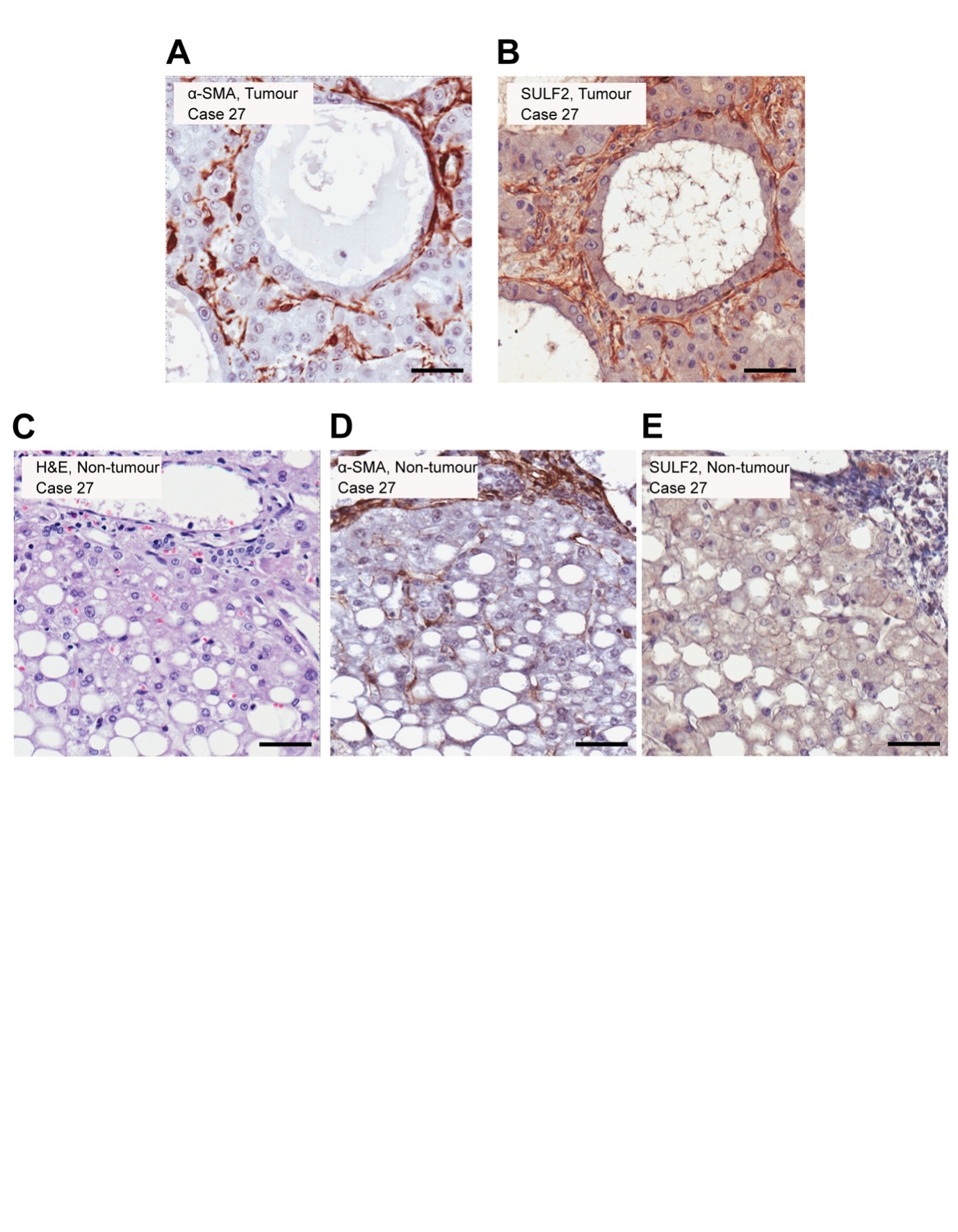


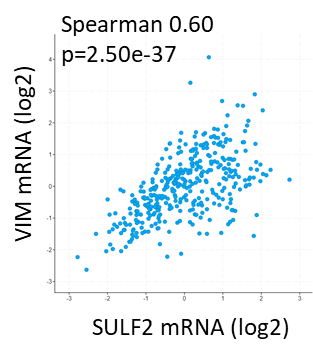

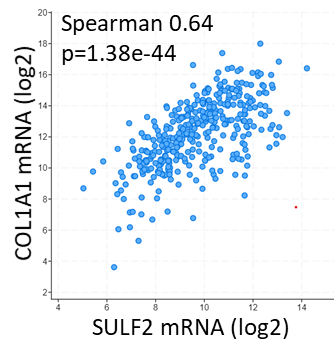

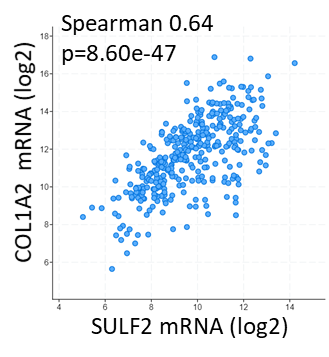

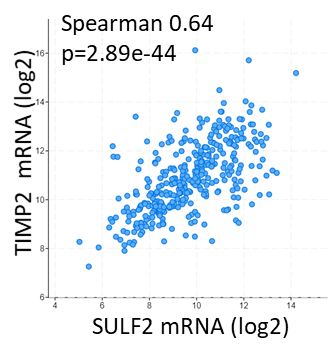


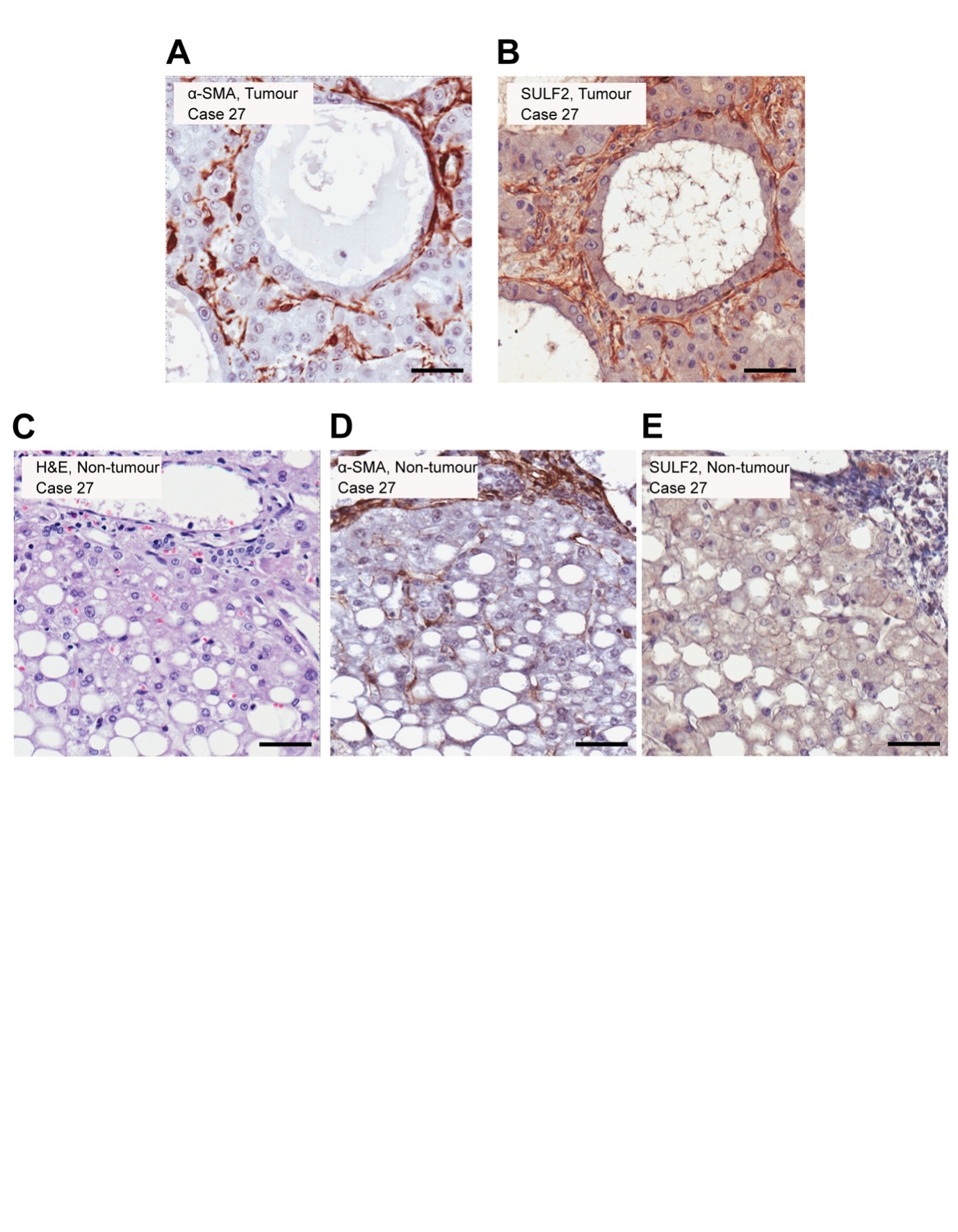
**C**

**Supplementary Figure 1: Expression of SULF2 and αSMA in non-neoplastic non-alcoholic fatty liver and HCC.**  Representative images show immunohistochemical (IHC) staining of αSMA and SULF2 **(A)** in HCC biopsy tissue (case 27). SULF2 was predominantly expressed in αSMA positive CAFs. The Cancer Genome Atlas (TGCA) Human Liver Cancer Dataset (Firehose Legacy, 371 patients) correlations between SULF2 and myofibroblast markers α-SMA(ACTA2), Vimentin (VIM), collagens (COL1A1, COL1A2) and tissue inhibitor of metalloproteinase 2 (TIMP2) mRNA are shown in (**B**). In non-tumour liver **(C)**, αSMA positive myofibroblasts in sinusoidal spaces between fat laden hepatocytes did not express SULF2. Images were captured using AperioImagescope software at x20 magnification and scale bars equal 50 microns.

Supplementary Figure 2


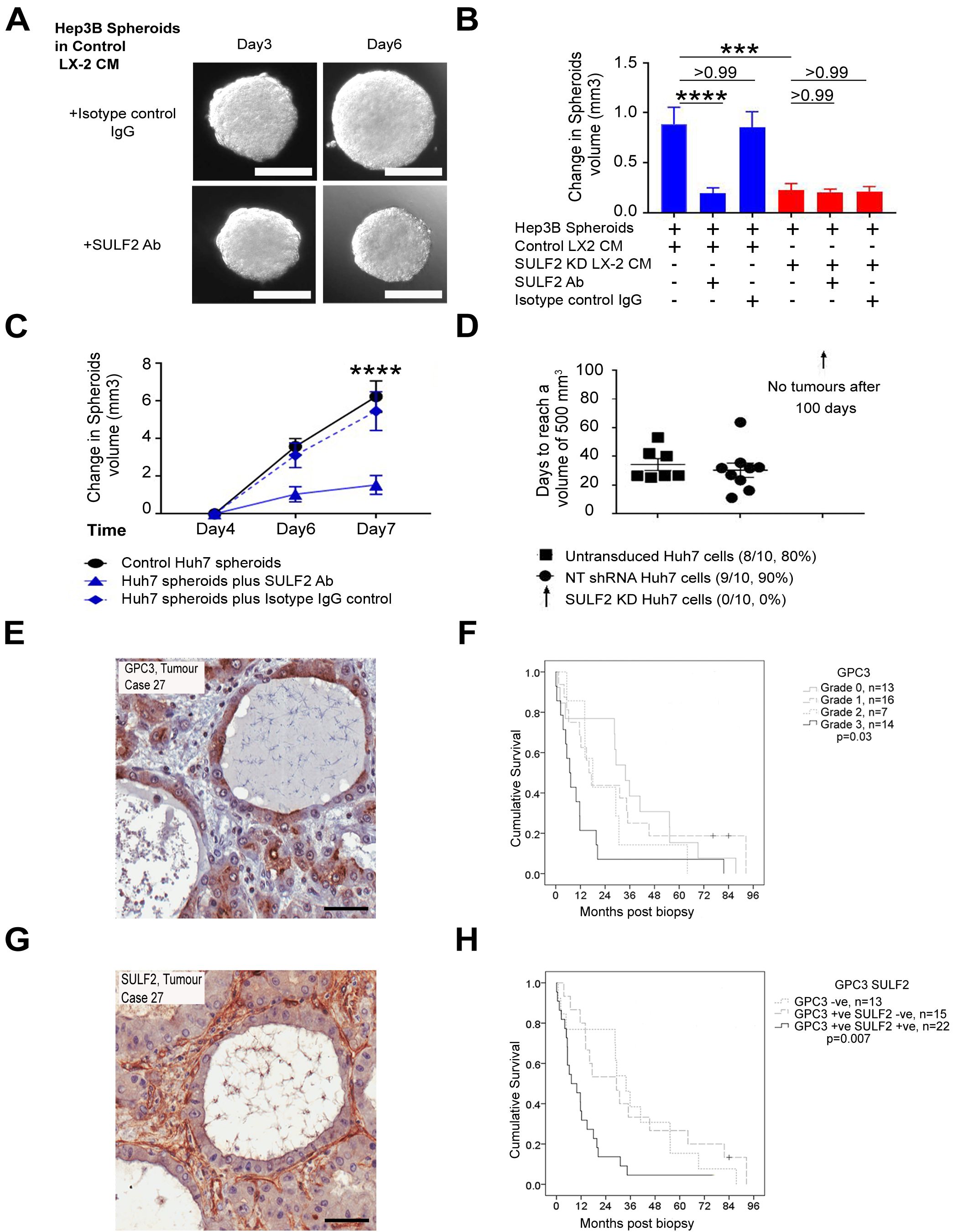

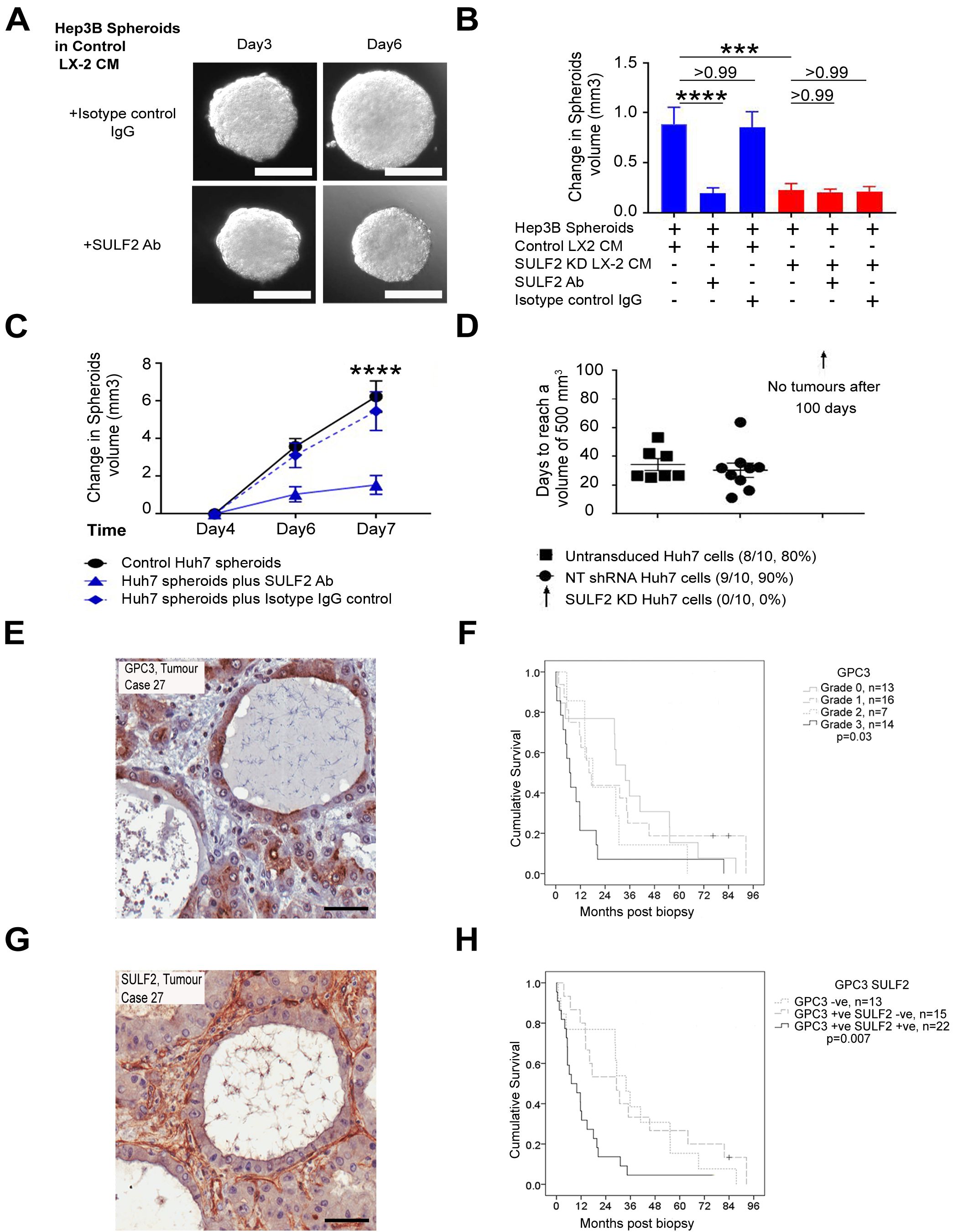


**A**

**B**

**C**

**D**


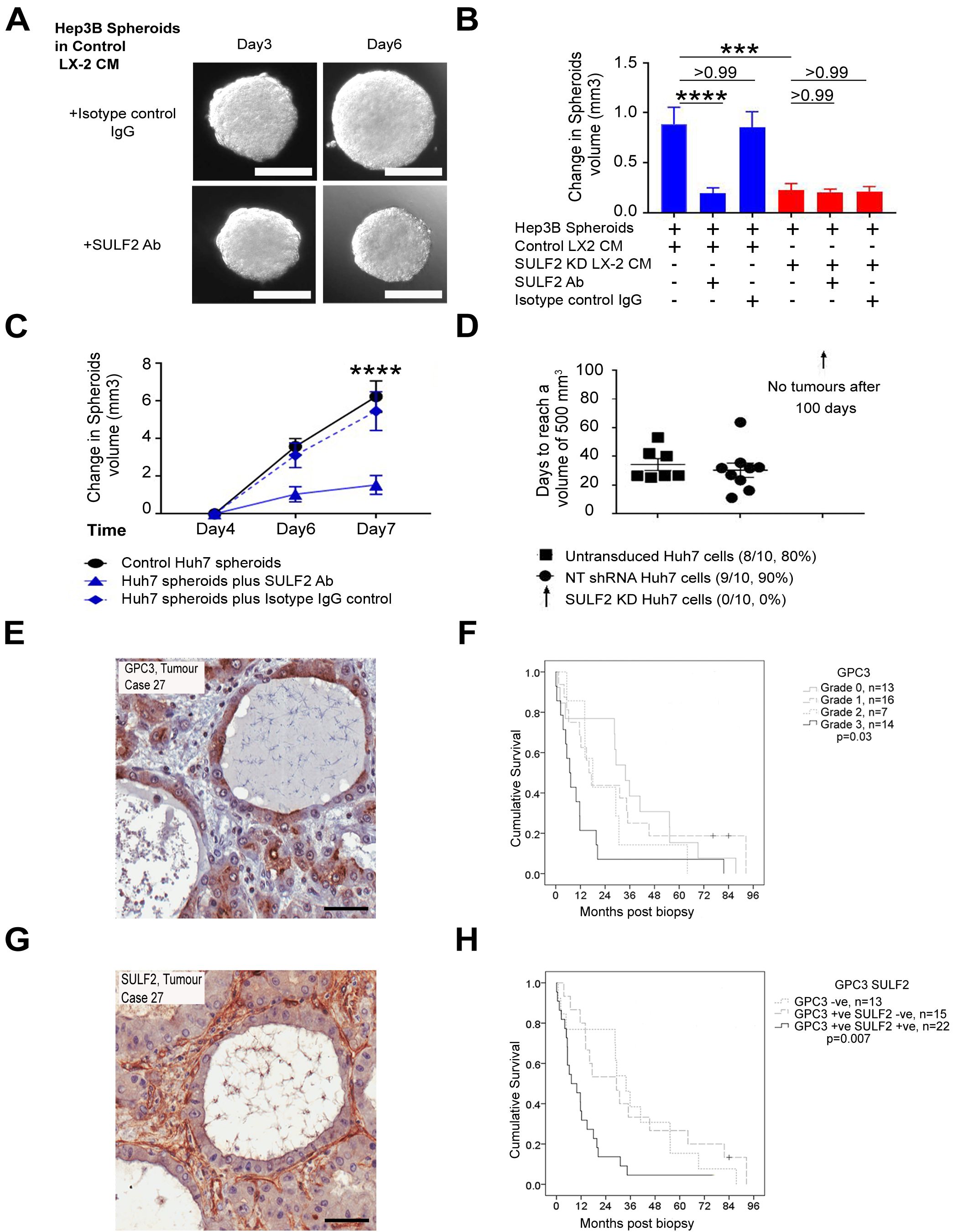

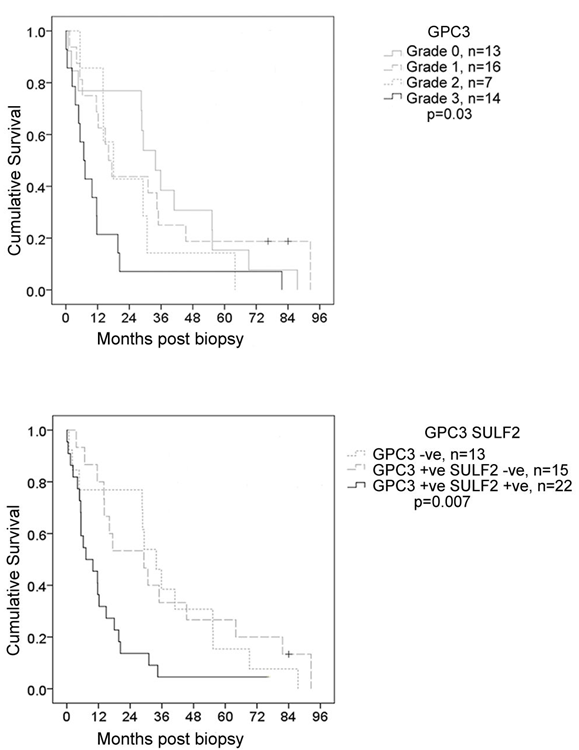

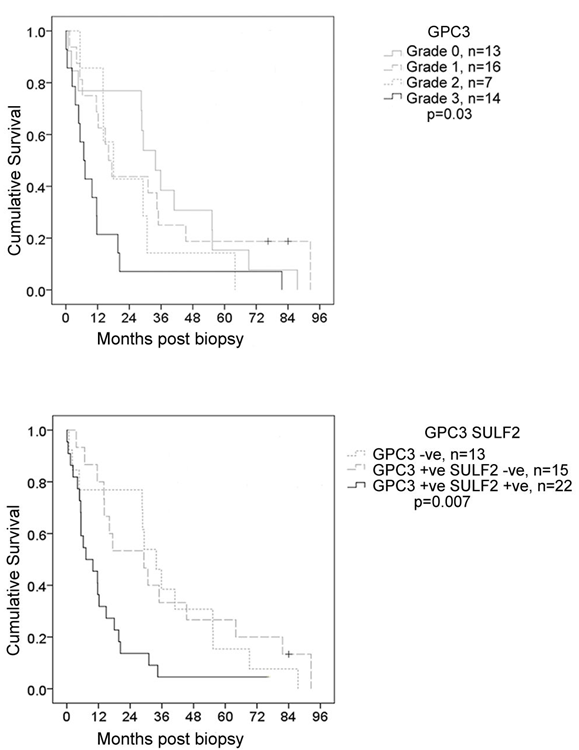


**Supplementary Figure 2: GPC3^+^/CAF SULF2^+^ HCC patients had the poorest prognosis**

Representative images show GPC3 immunohistochemical staining in the tumour of case 27 (**A**). Considering GPC3 as a single biomarker, the Kaplan Meier plot shows that grade 3 GPC3, present in 14 patients, distinguished patients with a significantly poorer survival (**B**). CAF SULF2 was present in adjacent tumour stroma in case 27 (**C**). The presence or absence of CAF SULF2 stratified the 23 patients with grades 1 and 2 GPC3 into two prognostic categories, where survival in the presence of CAF SULF2 was similar to that of GPC3 grade 3 cases and indistinguishable from the GPC3 absent patients in those with grade 1 or 2 GPC3 in the absence of stromal SULF2 (**D**). Scale bars are equivalent to 50 microns.

Supplementary Figure 3


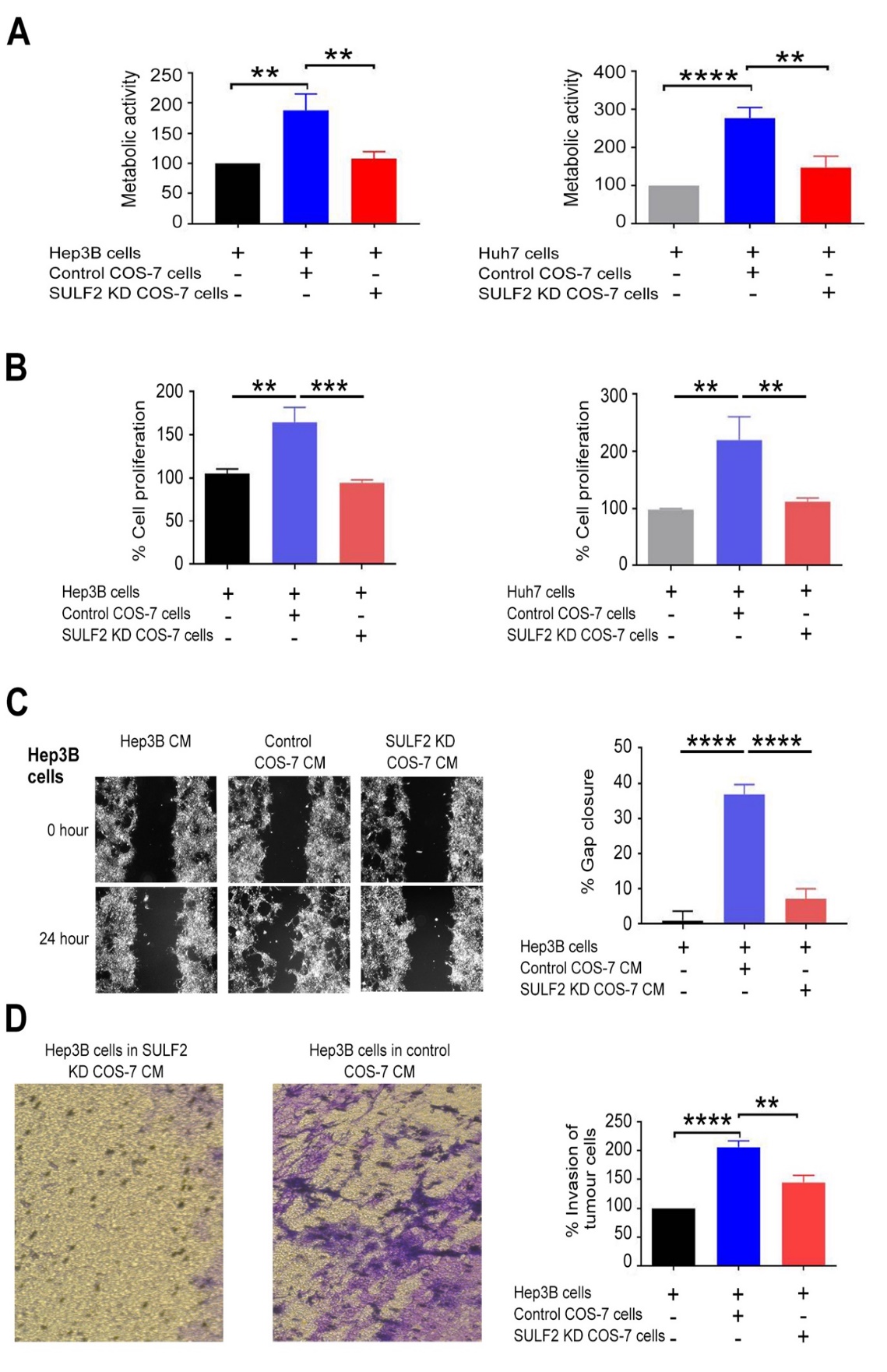

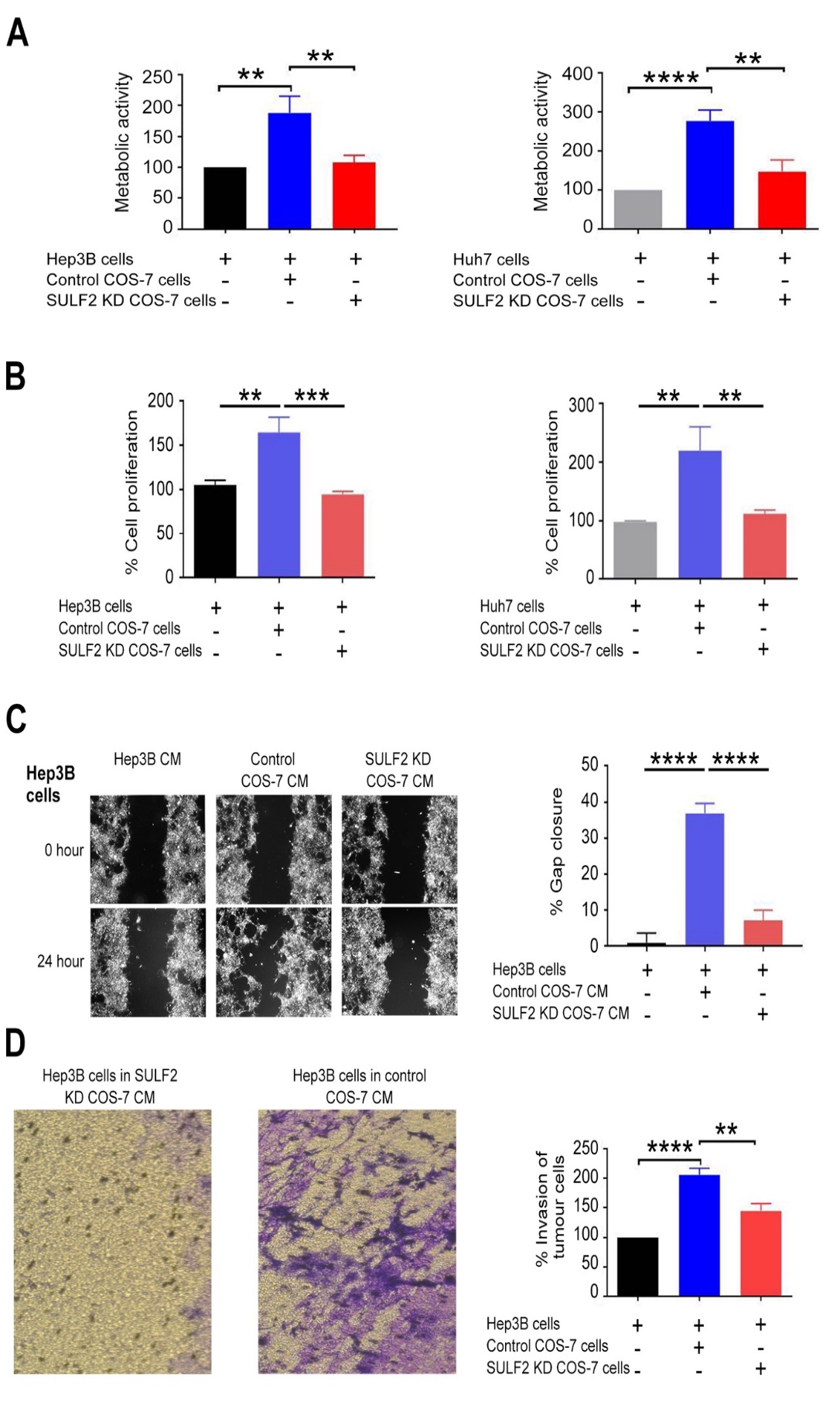


**A**


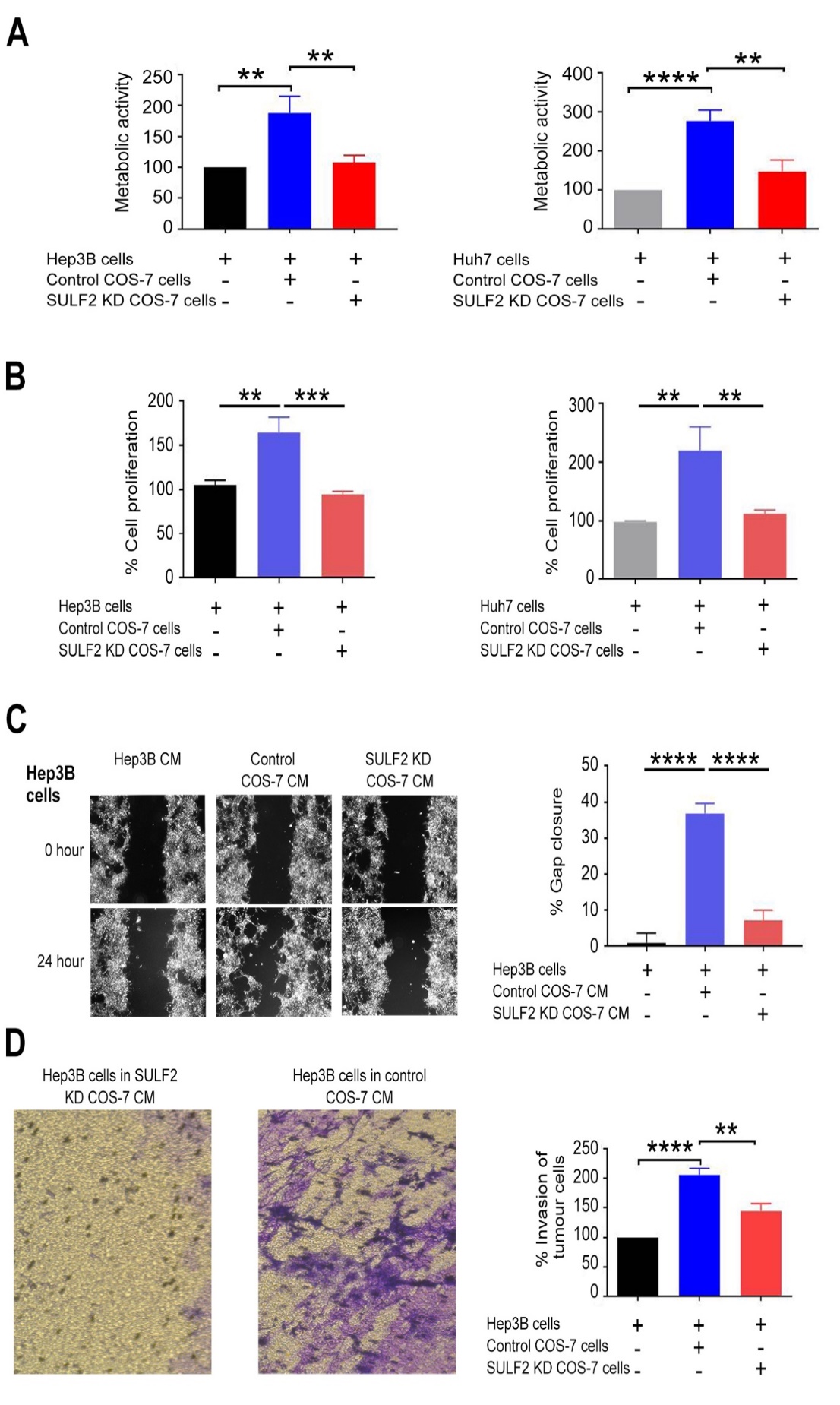

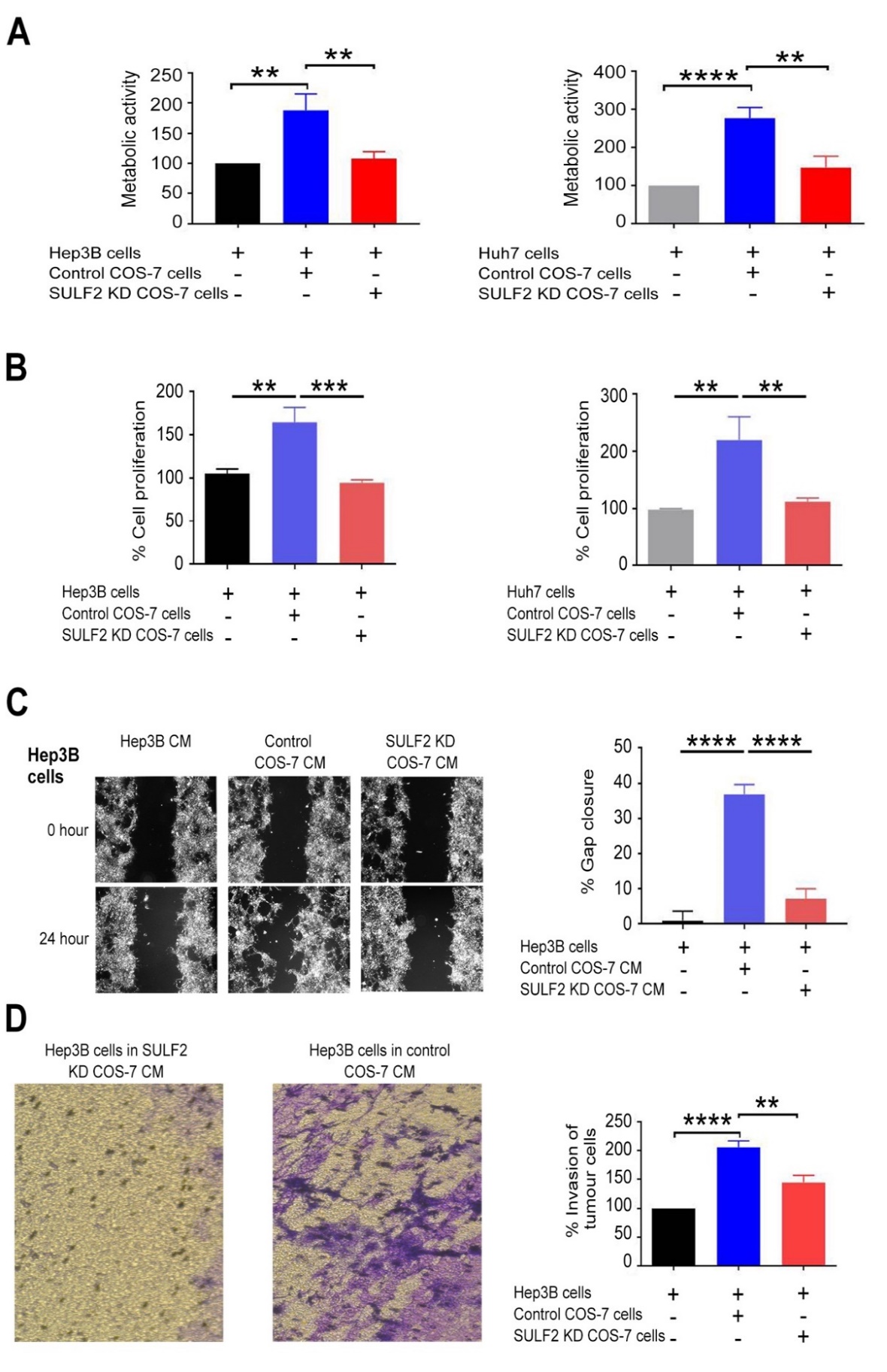


**BB**


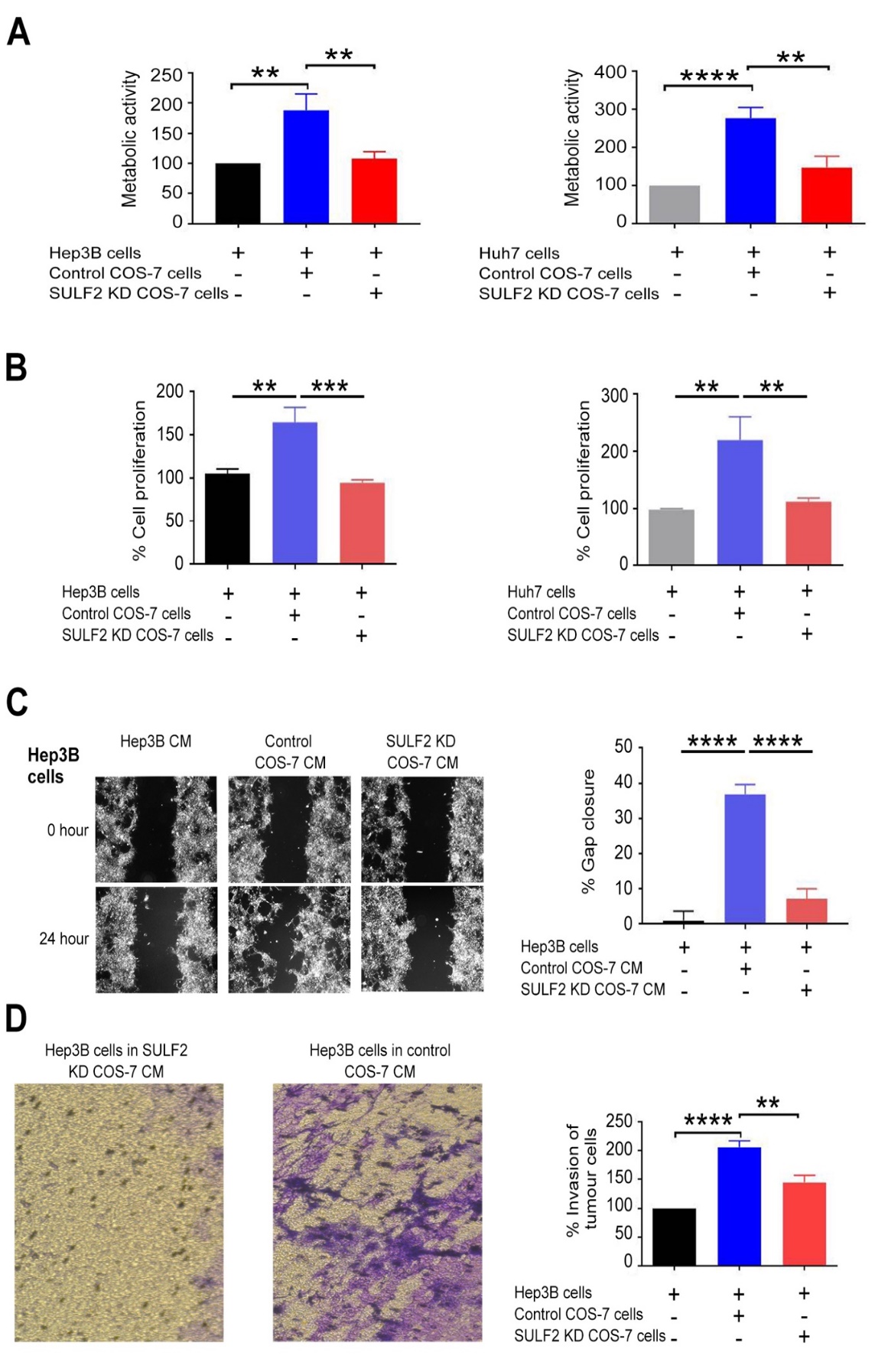

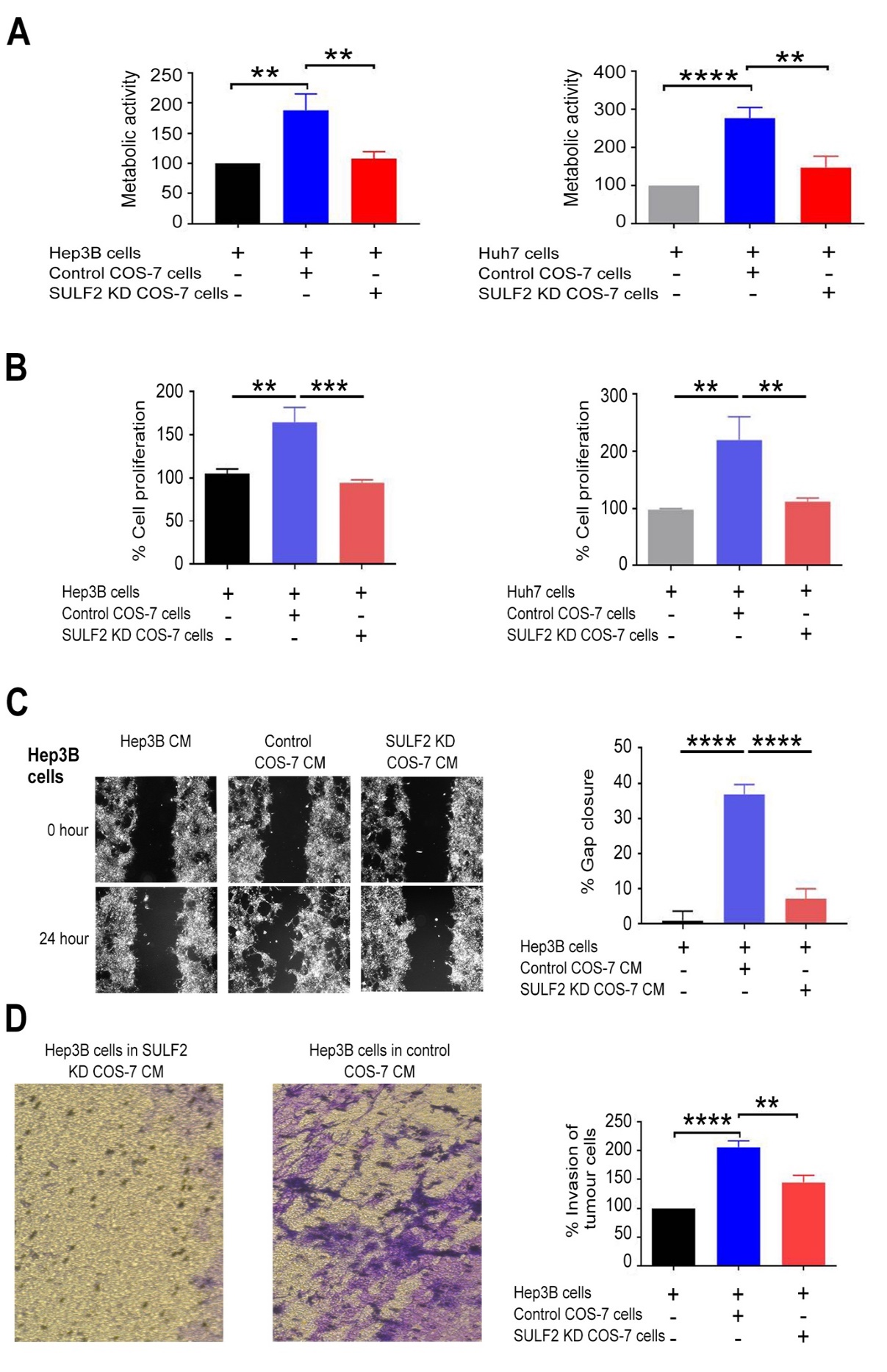


**C**


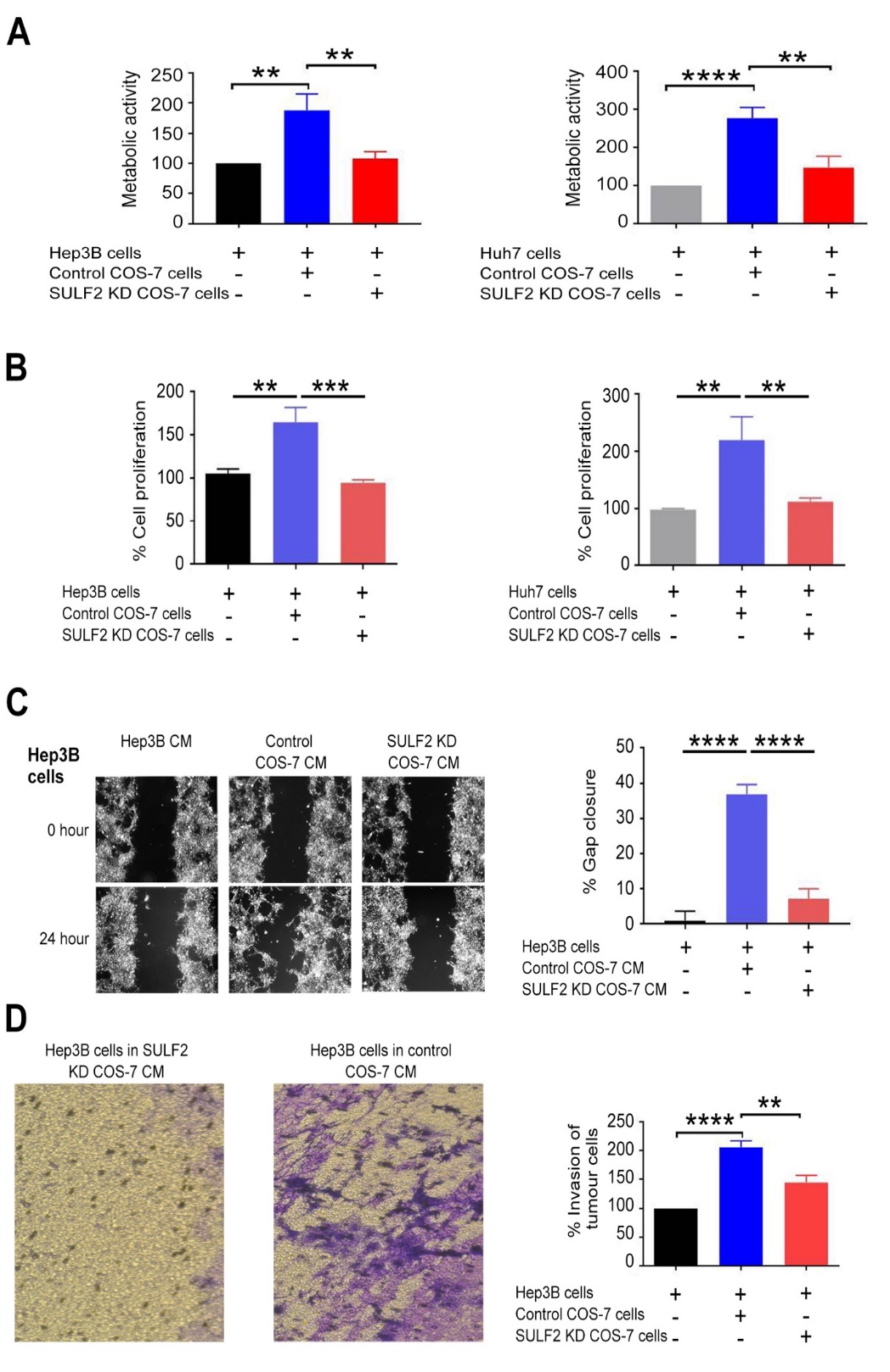

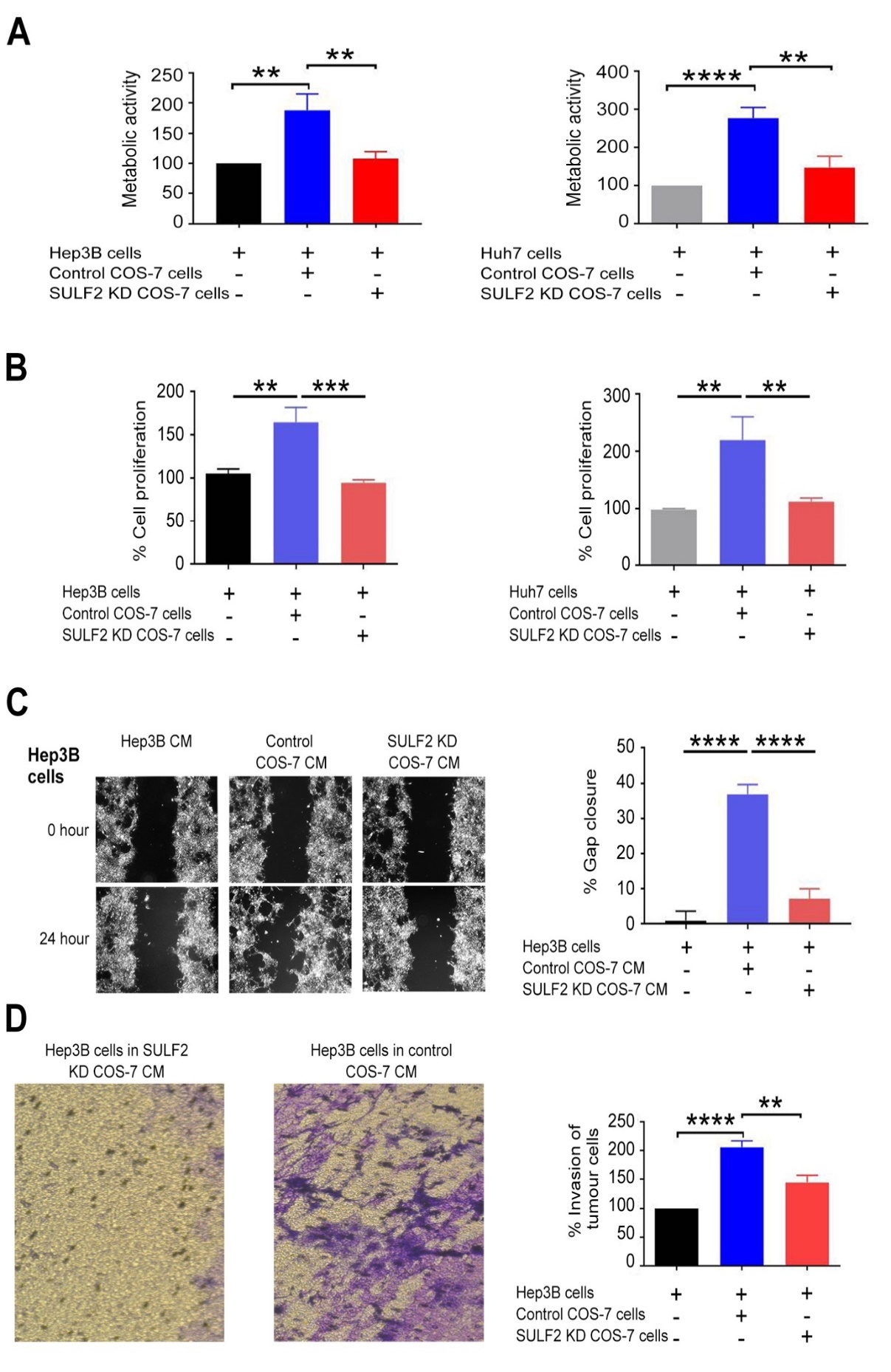


**D**

**Supplementary Figure 3: Stromal SULF2 stimulates the metabolic activity, cellular proliferation, migration and invasion of tumour cells.** Metabolic activity assessed by MTT assay in Hep3B (left) or Huh7 cells (right), +/- stromal SULF2 is shown in **A**. Cellular proliferation (BrDU incorporation) in Hep3B (left) or Huh7 cells (right) +/- stromal SULF2 is shown in **B**. Representative images, with quantification, of Hep3B cell migration in response to stromal CM from control COS-7 (SULF2) or SULF2 KD COS-7 (without SULF2) cells is in **C**. Data are mean ± s.e.m; n=3 independent experiments. Boyden chamber invasion shows COS-7 cell CM promoted Hep3B cell migration, as compared to Hep3B or SULF2 KD COS-7 cell CM, with representative images and quantification (mean ± s.e.m; n=5 independent experiments). **p= 0.01, ***p= 0.001, ****p= 0.0001.

Supplementary Figure 4


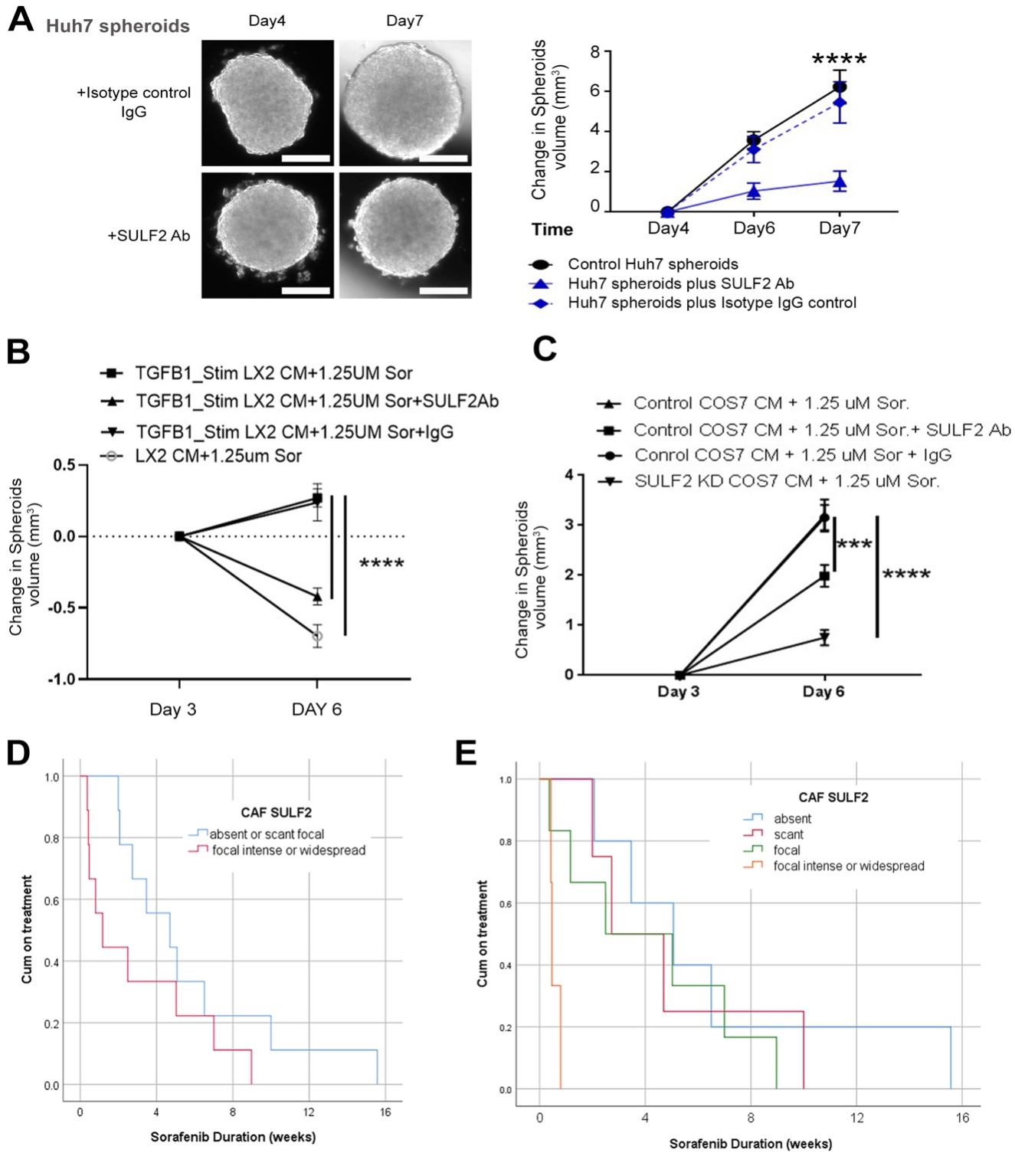


**A**

**C**

**B**


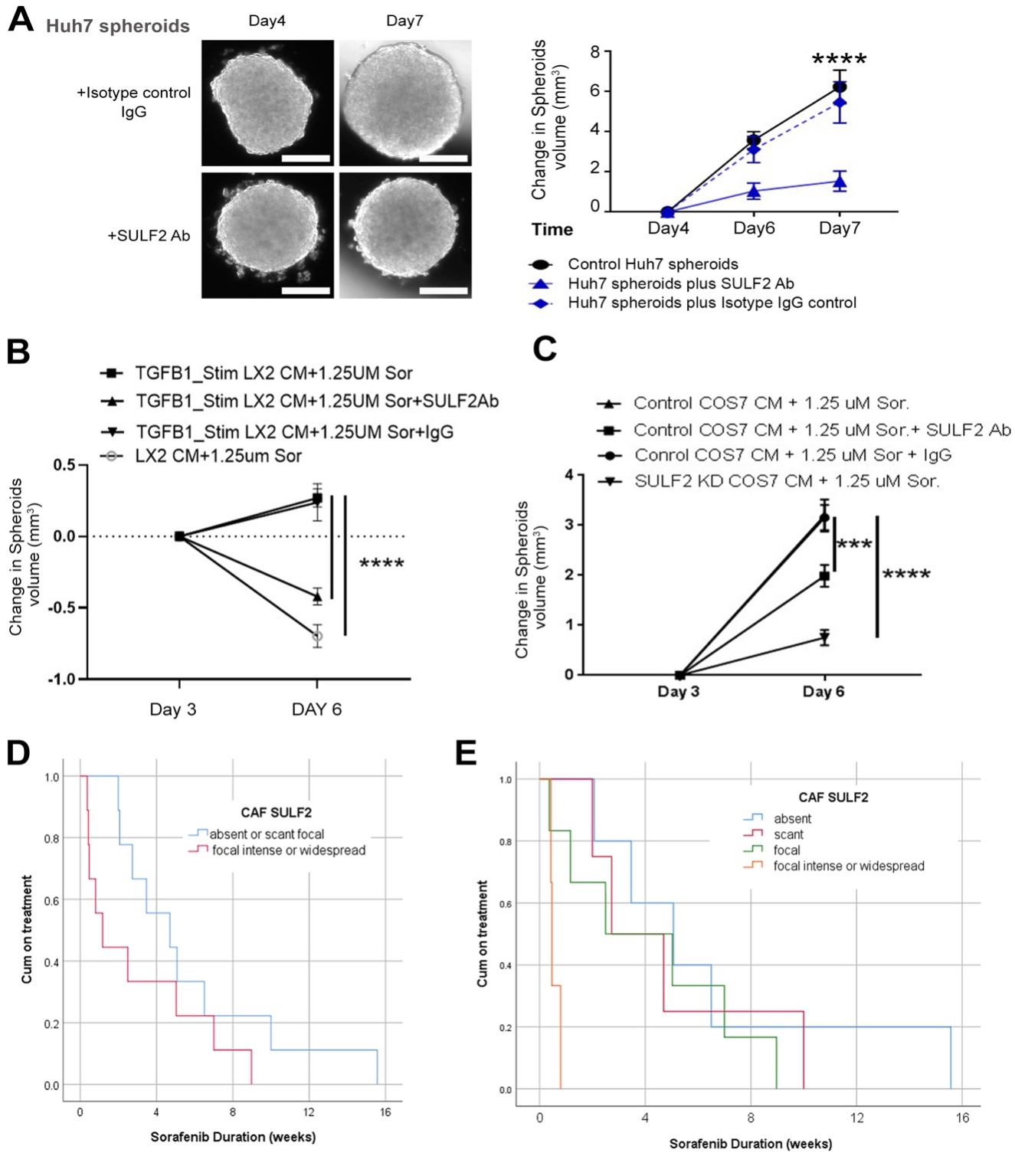
**Supplementary Figure 4: The impact SULF2 inhibition on the growth of tumour spheroids sorafenib sensitivity.** Growth of Huh7 spheroids (express endogenous SULF2), was abrogated using SULF2 antibody versus control IgG (**A**). Sorafenib treatment had little impact Hep3B spheroid growth cultured in TGFβ stimulated LX-2 cell CM (contains SULF2), with growth dramatically suppressed by sorafenib by the addition of SULF2 antibody (**B**). Sorafenib treatment had little impact on Hep3B spheroids growth in COS-7 cell CM (contains SULF2), with sensitivity rescued by the addition of SULF2 antibody (**C**). Duration of sorafenib therapy was reduced in patients with tumour stroma SULF2 compared to those with absent/scanty expression (median 29.2 months; Kaplan Meier, p=0.022)(**D**).

**D**

Supplementary Figure 5

**
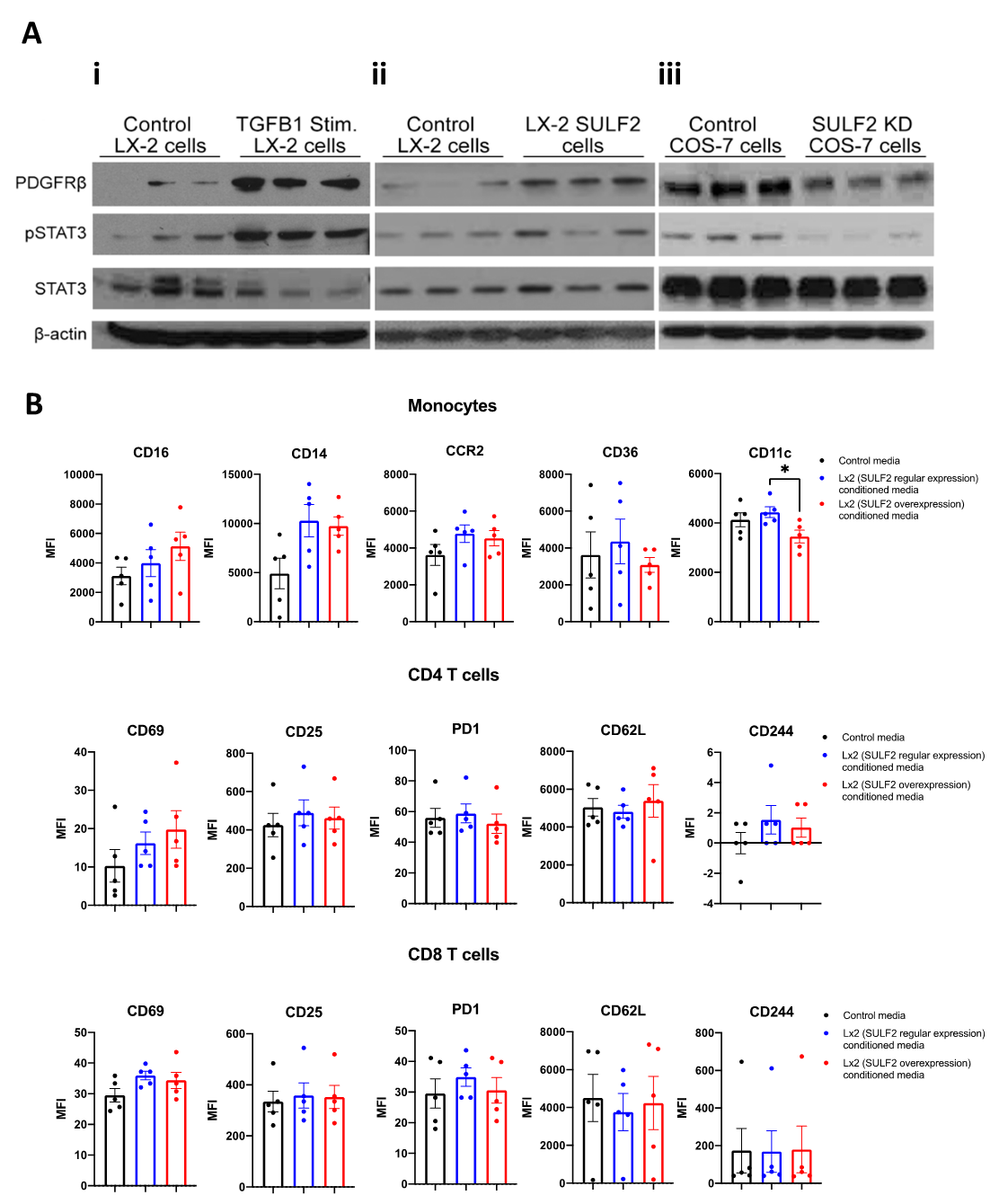
**

**Supplementary Figure 5: Stromal SULF2, induced by TGFβ, characterises an immune HCC phenotype.** Western blot shows SULF2-induced PDGFRβ expression and activation of the STAT3 pathway in LX-2 cells upon TGFβ stimulation **(Ai)** or following transfection with a SULF2 expression vector **(Aii)**. SULF2 KD in COS-7 cells was associated with reduced PDGFRβ expression and reduced STAT3 activation, compared to control COS-7 cells **(Aiii).** In PBMC culture experiments, SULF2 in CM impacted the phenotype of activated monocytes (See Figure 5 main manuscript), but did not alter this panel of markers of lymphocyte phenotype.

## Supplementary references

1. Edmondson HA, Steiner PE. Primary carcinoma of the liver: a study of 100 cases among 48,900 necropsies. Cancer 1954;7:462-503.

2. EASL-EORTC. European Association for the Study of the Liver, European Organisation for Research and Treatment of Cancer: EASL-EORTC clinical practice guidelines: management of hepatocellular carcinoma. J Hepatol 2012;56:43.
